# Unraveling the Complex Solid-State Phase Transition Behavior of 1-Iodoadamantane, a Material for Which Ostensibly Identical Crystals Undergo Different Transformation Pathways

**DOI:** 10.1021/acs.cgd.3c00223

**Published:** 2023-04-12

**Authors:** Okba Al Rahal, Benson M. Kariuki, Colan E. Hughes, P. Andrew Williams, Xiaoyan Xu, Simon Gaisford, Dinu Iuga, Kenneth D. M. Harris

**Affiliations:** † School of Chemistry, Cardiff University, Park Place, Cardiff, Wales CF10 3AT, U.K.; ‡ Department of Pharmaceutics, School of Pharmacy, University College London, 29-39 Brunswick Square, London, England WC1N 1AX, U.K.; § Department of Physics, University of Warwick, Coventry CV4 7AL, England, U.K.

## Abstract

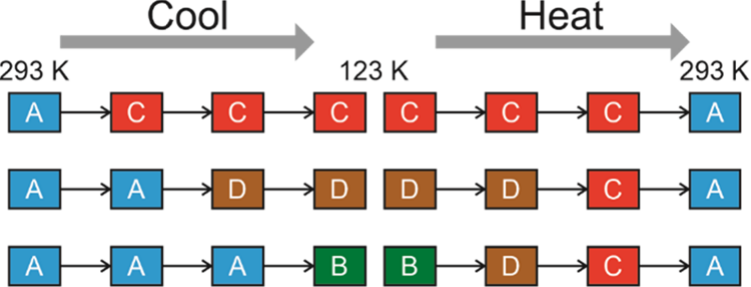

Phase transitions in crystalline molecular solids have important implications in the fundamental understanding of materials properties and in the development of materials applications. Herein, we report the solid-state phase transition behavior of 1-iodoadamantane (1-IA) investigated using a multi-technique strategy [synchrotron powder X-ray diffraction (XRD), single-crystal XRD, solid-state NMR, and differential scanning calorimetry (DSC)], which reveals complex phase transition behavior on cooling from ambient temperature to ca. 123 K and on subsequent heating to the melting temperature (348 K). Starting from the known phase of 1-IA at ambient temperature (phase **A**), three low-temperature phases are identified (phases **B**, **C**, and **D**); the crystal structures of phases **B** and **C** are reported, together with a re-determination of the structure of phase **A**. Remarkably, single-crystal XRD shows that some individual crystals of phase **A** transform to phase **B**, while other crystals of phase **A** transform instead to phase **C**. Results (from powder XRD and DSC) on cooling a powder sample of phase **A** are fully consistent with this behavior while also revealing an additional transformation pathway from phase **A** to phase **D**. Thus, on cooling, a powder sample of phase **A** transforms partially to phase **C** (at 229 K), partially to phase **D** (at 226 K) and partially to phase **B** (at 211 K). During the cooling process, each of the phases **B**, **C**, and **D** is formed *directly* from phase **A**, and no transformations are observed *between* phases **B**, **C**, and **D**. On heating the resulting triphasic powder sample of phases **B**, **C**, and **D** from 123 K, phase **B** transforms to phase **D** (at 211 K), followed by the transformation of phase **D** to phase **C** (at 255 K), and finally, phase **C** transforms to phase **A** (at 284 K). From these observations, it is apparent that different crystals of phase **A**, which are ostensibly identical at the level of information revealed by XRD, must actually differ in other aspects that significantly influence their low-temperature phase transition pathways. This unusual behavior will stimulate future studies to gain deeper insights into the specific properties that control the phase transition pathways in individual crystals of this material.

## Abstract 2

We report the complex solid-state phase transition behavior of 1-iodoadamantane, investigated using a multi-technique strategy, including the identification and characterization of three low-temperature phases. Remarkably, it is found that different crystals of the known phase at ambient temperature, which are ostensibly identical at the level of information revealed by X-ray diffraction, can exhibit different low-temperature phase transition pathways.

## 1 Introduction

**Figure 1:**
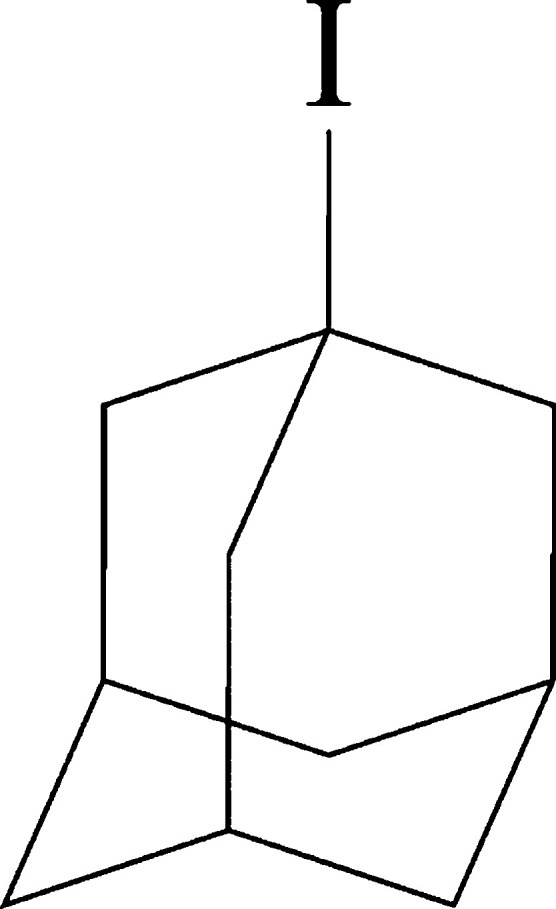
Molecular structure of 1-IA.

Phase transition behavior in crystalline molecular solids has long been of interest,^1,2^ motivated by the quest to determine the changes in structural and dynamic properties associated with phase transitions through experimental observations, to derive theoretical frameworks for rationalizing the nature of phase transitions, and to exploit the changes in physical properties that occur at phase transitions as the basis of materials applications. In recent years, there has been renewed interest in the phase transition behavior and temperature-dependent structural properties of members of the family of 1-halogenoadamantanes, in particular 1-fluoroadamantane,^3−7^ 1-chloroadamantane,^8−14^ 1-bromoadamantane^13−17^ and solid solutions containing 1-halogenoadamantanes, in particular the 1-chloroadamantane/1-cyanoadamantane^18−20^ and 1-bromoadamantane/1-chloroadamantane^21^ systems. However, significantly less attention has been devoted to 1-iodoadamantane (1-IA; Figure 1). To address this issue, the present paper reports the solid-state phase transition behavior and temperature-dependent structural properties of 1-IA, focusing on the regime of cooling from ambient temperature to ca. 123 K, followed by heating to the melting temperature.

Although it was reported in 1983 by Virlet et al.^22^ that 1-IA is a rotator phase solid at ambient temperature, based primarily on solid-state NMR data, the phase transition behavior and the low-temperature structural properties were not investigated in detail. An earlier study of the thermal properties of various derivatives of adamantane by Clark et al.^23^ reported that 1-IA undergoes a low-temperature phase transition at 211 K and has a melting temperature of 347 K. In 1988, Foulon and Gors^24^ reported the crystal structure of 1-IA at 295 and 256 K, both of which represent the high-temperature phase of 1-IA (denoted phase **A** in the present paper). In the reported structure,^24^ each 1-IA molecule is disordered between two orientations related by 60° rotation about the molecular 3-fold symmetry axis (the C–I bond axis). However, a re-determination of the crystal structure of the high-temperature phase **A** in the present paper leads instead to the conclusion that the time-averaged and space-averaged structure, as determined by single-crystal X-ray diffraction (XRD), is actually an ordered structural model, as discussed in Section 2.5.1. Previous solid-state NMR studies of the temperature dependence of ^1^H NMR relaxation^22^ relied heavily on the structural descriptions from the work of Foulon and Gors^24^ to interpret their data for phase **A**, and also reported the occurrence of a phase transition at ca. 211 K on cooling. However, in the absence of knowledge of the low-temperature crystal structure(s), the solid-state NMR data below 211 K were interpreted only at a qualitative level.

In the present work, the phase transition behavior and temperature-dependent structural properties of 1-IA have been studied by single-crystal XRD, powder XRD, and differential scanning calorimetry (DSC) on cooling samples of phase **A** from ambient temperature to low temperature (120 K for single-crystal XRD and 123 K for powder XRD). The powder XRD and DSC studies also investigated the behavior on subsequent heating to the melting temperature. In addition, insights on the dynamic nature of the 1-IA molecules in phase **A** have been established from high-resolution solid-state ^13^C NMR spectroscopy. The results of these studies reveal complex phase transition behavior, both on cooling and subsequent heating, involving three distinct low-temperature phases and differences in the phase transition pathways observed on the cooling and heating cycles. Remarkably, it is found that crystals of phase **A** that are ostensibly identical based on XRD studies can exhibit three different types of low-temperature phase transition behavior, leading to a different low-temperature phase in each case.

## 2 Results and Discussion

### 2.1 Sample Characterization

All samples of 1-IA studied in this work were crystallized from ethanol. For all crystallized samples, the experimental powder XRD data at ambient temperature matched the simulated powder XRD data for the crystal structure of phase **A** reported in Section 2.5.1, confirming that all crystallized samples were monophasic samples of phase **A**.

### 2.2 Phase Transition Behavior from DSC Studies

**Figure 2:**
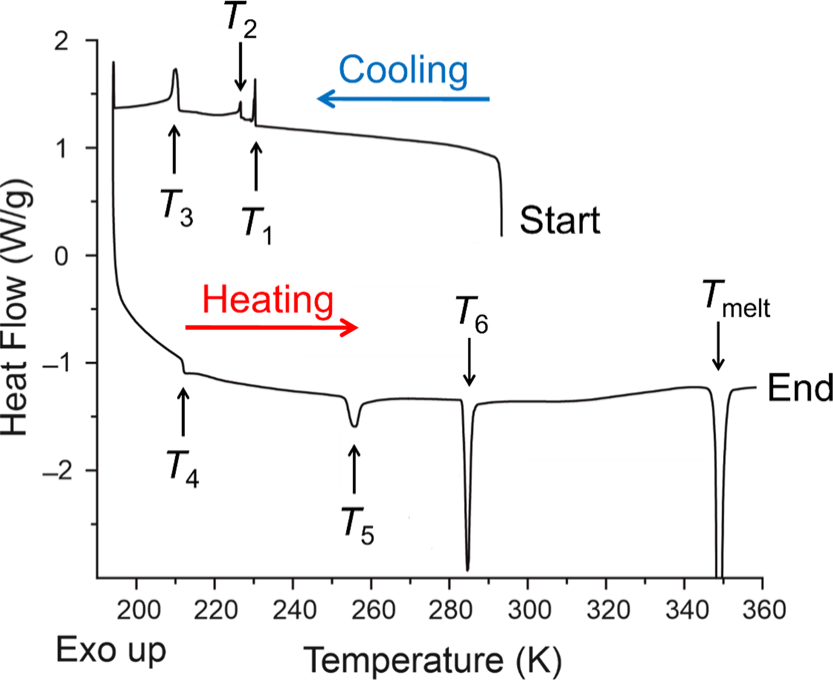
DSC data recorded for 1-IA as a function of temperature on cooling from ambient temperature, followed by heating to the melting temperature (with cooling and heating rates of 5 K min^–1^). The peak due to melting is truncated.

A representative DSC dataset recorded for 1-IA on cooling from phase **A** at ambient temperature to 190 K and on subsequent heating to the melting temperature (at cooling/heating rates of 5 K min^–1^) is shown in Figure 2. In the cooling cycle, exothermic events are observed at *T*_1_ ≈ 229 K, *T*_2_ ≈ 226 K, and *T*_3_ ≈ 211 K and are assigned, in conjunction with results from single-crystal XRD and powder XRD discussed below, as solid–solid phase transitions. On subsequent heating from 190 K, endothermic events are observed at *T*_4_ ≈ 211 K, *T*_5_ ≈ 255 K, and *T*_6_ ≈ 284 K and are also assigned as solid–solid phase transitions. A large endothermic peak at *T*_melt_ ≈ 348 K is attributed to melting.

To assess the reproducibility of the DSC results, a total of nine independent DSC measurements were made, involving two different batches of 1-IA prepared by the same crystallization procedure and giving identical powder XRD data at ambient temperature characteristic of phase **A**. For each of these batches of 1-IA, two different cooling/heating rates (5 and 20 K min^–1^) were used. Examples of these DSC data are shown in Figure S1 (for the same batch of 1-IA used to record the DSC data in Figure 2, but using a cooling/heating rate of 20 K min^–1^) and Figure S2 (for the other batch of 1-IA and using the same cooling/heating rate (5 K min^–1^) used to record the DSC data in Figure 2). All nine DSC datasets showed clear and well-defined events corresponding to *T*_1_, *T*_3_, *T*_4_, *T*_6_, and *T*_melt_. However, one dataset lacked a clear event corresponding to *T*_2_ and one dataset lacked clear events corresponding to *T*_2_ and *T*_5_. The onset temperatures were measured using the TA Universal Analysis software. The mean onset temperature for each event (in each case taking the mean of the values of onset temperature from all datasets in which the event was clearly observed) and the standard deviation in the mean onset temperature (shown in parentheses) are as follows: <*T*_1_> = 229.1 K (1.7 K), <*T*_2_> = 226.1 K (1.7 K), <*T*_3_> = 210.7 K (0.2 K), <*T*_4_> = 211.5 K (0.3 K), <*T*_5_> = 255.1 K (1.4 K), <*T*_6_> = 283.6 K (0.9 K), <*T*_melt_> = 348.4 K (0.1 K). We note that the events corresponding to *T*_3_, *T*_4_, and *T*_melt_ show greater reproducibility (i.e., lower standard deviation) in the onset temperature.

### 2.3 Low-Temperature Single-Crystal XRD

Single-crystal XRD studies were carried out to investigate the structural changes associated with the thermal events observed at low temperature in the DSC data, involving independent studies of eight different single crystals on cooling. Single-crystal XRD measurements were started above 230 K and involved either brief data collections for unit cell determination or full data collections for structure determination at several temperatures on cooling. In each experiment, the lowest temperature studied was in the range between 210 and 120 K (specific details for each experiment are given in Supporting Information; Table S1). In each experiment, the unit cell and/or crystal structure determined from the single-crystal XRD data indicated that the crystal was in phase **A** at the starting temperature (above 230 K). In all cases for which crystal structure determination of phase **A** was carried out, the crystal structure was identical within experimental errors.

However, on cooling the eight crystals, two distinct types of behavior were observed. For behavior (i), six crystals of phase **A** were found to undergo a structural change at ca. 210 K to a new phase designated as phase **B** (corresponding to the transition at *T*_3_ ≈ 211 K in our DSC data and corresponding to the low-temperature phase transition reported previously from thermal analysis^23^ and solid-state NMR^22^ studies). For behavior (ii), two crystals of phase **A** were found to undergo a structural change at ca. 230 K (corresponding to *T*_1_ ≈ 229 K in our DSC data) to another new phase designated as phase **C**. We note that none of the crystals studied showed a structural change at ca. 226 K (corresponding to *T*_2_ in our DSC data), and none of the single crystals was observed to undergo more than one phase transition within the temperature range investigated (the lowest temperatures studied were 120 K for phase **B** and 175 K for phase **C**; see Table S1).

The temperatures at which the structural changes occur for behavior (i) and behavior (ii) are close to the temperatures of the exothermic events labeled *T*_3_ and *T*_1_, respectively, in the cooling cycle of our DSC data (Figure 2). The transformation from phase **A** to phase **B** in behavior (i) and the transformation from phase **A** to phase **C** in behavior (ii) both occurred with retention of sufficient crystal quality to allow the crystal structures of phase **B** and phase **C** to be determined from single-crystal XRD data. Crystallographic data for phases **A**, **B**, and **C** are given in Table 1, and the crystal structures are discussed in Section 2.5.

**Table 1:** Crystallographic Data for Phases **A**, **B**, and **C** of 1-IA^a^

	phase **A**	phase **B**	phase **C**
*T*/K	290(2)	175(2)	230(2)
*F*_w_/g mol^–1^	262.12	262.12	262.12
crystal system	orthorhombic	monoclinic	monoclinic
space group	*Pm*2_1_*n*	*P*2_1_	*P*2_1_/*c*
λ/Å	0.71073	0.71073	0.71073
crystal size/mm^3^	0.178 × 0.151 × 0.098	0.374 × 0.250 × 0.200	0.780 × 0.466 × 0.237
*a*/Å	6.6977(12)	6.5776(9)	10.278(3)
*b*/Å	8.6709(12)	8.5074(10)	6.9402(16)
*c*/Å	8.8476(11)	8.8193(10)	13.826(3)
α/°	90	90	90
β/°	90	94.482(12)	90.00(2)
γ/°	90	90	90
*V*/Å^3^	513.83(13)	492.00(11)	986.2(4)
density/g cm^–3^	1.694	1.769	1.765
*Z*	2	2	4

a The structure of phase **A** is described in space group *Pm*2_1_*n*, rather than the conventional *Pmn*2_1_ setting for this space group, in order to facilitate comparison to the structure of phase **B**.

The six crystals exhibiting behavior (i) did not undergo any transition at ca. 230 K but remained as phase **A** upon cooling close to 210 K (the period of time at which these crystals were at temperatures between 230 and 210 K was up to 30 min). Two of these crystals (crystals **1** and **2** in Table S1) were studied at a temperature just above 210 K (220 K for crystal **1**; 215 K for crystal **2**) and were still in phase **A**, but single-crystal XRD data recorded at 210 K showed that these crystals had transformed to phase **B**. The evolution of unit cell volume as a function of temperature for these two crystals on cooling (Figure S3) shows an abrupt decrease in volume at ca. 210 K due to the transformation from phase **A** to phase **B**. For the other four crystals exhibiting behavior (i), single-crystal XRD data were not recorded at 210 K; however, at the first temperature at which data were recorded below 210 K, these crystals had transformed to phase **B**. The crystal structure of phase **B** is discussed in Section 2.5.3.

For the two crystals exhibiting behavior (ii), the crystal was cooled from 280 to 230 K (at 6 K min^–1^) and then held at 230 K, while single-crystal XRD data were recorded. For one crystal (crystal **7** in Table S1), the data recorded immediately on reaching 230 K showed that the crystal had transformed to phase **C** before starting the data collection (clearly, the specific temperature between 280 and 230 K at which this crystal transformed to phase **C** was not established in this experiment). For the other crystal (crystal **8** in Table S1), single-crystal XRD data recorded at 230 K showed that the crystal was still in phase **A** at the start of the data collection, but the crystal was observed to change from phase **A** to phase **C***during* the data collection; the structural change commenced ca. 4.5 min after starting the data collection (the total data collection time was 10 min) and co-existence of phases **A** and **C** within the crystal was observed for ca. 15 s (a diffraction image recorded during the period of co-existence of phases **A** and **C** in this crystal at 230 K is shown in Figure S4). Thus, it is clear that this crystal transformed from phase **A** to phase **C** at 230 K, but only following an induction period after reaching this temperature. The unit cell volume determined as a function of temperature on cooling this crystal is shown in Figure S5. From the observations for the two crystals that exhibited behavior (ii), there appears to be some variance in the temperature at which the transition from phase **A** to phase **C** occurs for different crystals. For both of these crystals, after transforming to phase **C**, no further structural transformations occurred on cooling to 175 K. The crystal structure of phase **C** is discussed in Section 2.5.4.

In all our single-crystal XRD studies, phase **A** was never observed to exist below 211 K (the temperature of the phase transition from phase **A** to phase **B**).

### 2.4 Variable-Temperature Synchrotron Powder XRD

#### 2.4.1 Overview

In order to establish the structural changes that occur in a polycrystalline sample of 1-IA as a function of temperature, and thus to correlate structural changes with thermal events observed on cooling and subsequently heating a polycrystalline sample of 1-IA in our DSC data (Figure 2), variable-temperature synchrotron powder XRD data were recorded on beamline I11 at Diamond Light Source. In our analysis of the powder XRD data, knowledge of the crystal structures of phases **A**, **B**, and **C** determined from our single-crystal XRD studies was crucial to allow identification of the specific phase(s) present in the powder sample at each temperature. In order to minimize the total time of exposure of the sample to the X-ray beam in the variable-temperature powder XRD experiment (recognizing that iodinated organic materials are susceptible to X-ray beam damage), the powder XRD data were recorded at increments of 10 K.

To facilitate the interpretation of the variable-temperature powder XRD data, the key “diagnostic peaks” that allow each phase of 1-IA to be uniquely identified are listed in Table 2. While our discussion of the phase(s) present at each temperature in the powder XRD study is based primarily on highlighting the presence or absence of these diagnostic peaks, we emphasize that our interpretations of the powder XRD data involved detailed inspection of the whole powder XRD pattern recorded at each temperature.

**Table 2:** Diagnostic Peaks in Powder XRD Data (Recorded at λ = 0.82462 Å) for Phases **A**, **B**, **C**, and **D** of 1-IA, Used in the Identification of Phases Present at Each Temperature in the Variable-Temperature Powder XRD Study^a^

phase	diagnostic peaks	*T*/K
**A**	2θ = 5.33°, 12.84°	213
**B**	2θ = 10.26°	203
**C**	2θ = 6.88°, 9.70°, 11.45°	213
**D**	2θ = 7.98°, 8.09°, 11.54°	213

a The temperature (*T*) refers to the specific temperature at which the quoted peak positions were measured in the powder XRD data. The temperatures were chosen to span as narrow a range as possible for the different phases and to be close to the center of the range of temperatures in the variable-temperature powder XRD study.

#### 2.4.2 Evolution of Solid Phases on Cooling

**Figure 3:**
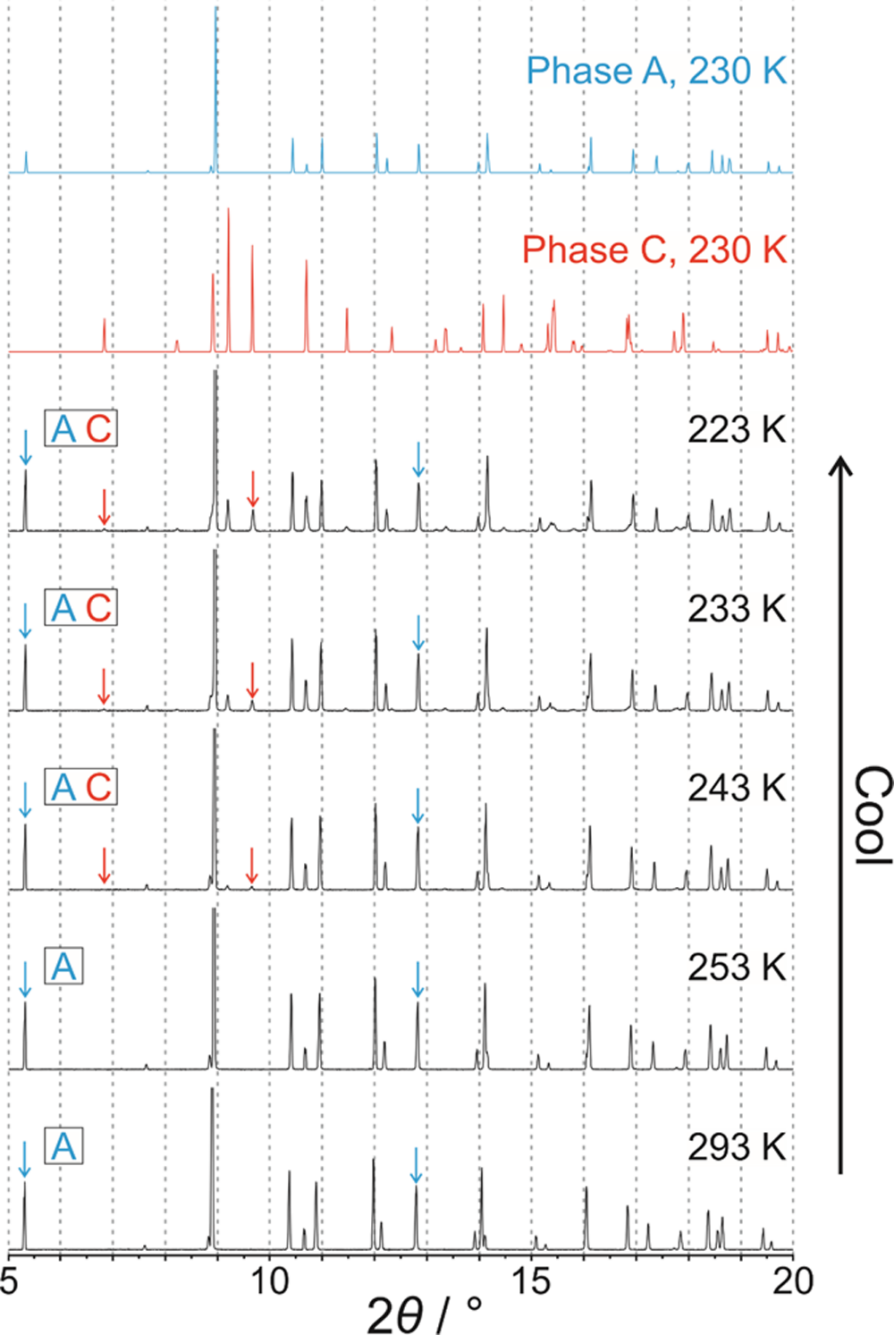
Powder XRD data recorded for 1-IA on cooling from 293 to 223 K. Peaks indicated by arrows are diagnostic peaks for phase **A** (cyan arrows) and phase **C** (red arrows). Powder XRD patterns simulated from the crystal structures of phase **A** (cyan) and phase **C** (red) are also shown. The phases present at each temperature are indicated at the left side.

The powder XRD data at 293 K (Figure 3) confirm that the polycrystalline sample at the start of our variable-temperature powder XRD study was a monophasic sample of phase **A**. From 293 to 253 K, the only changes observed in the powder XRD data are peak shifts attributed to lattice contraction of phase **A** upon cooling (the powder XRD data recorded at all temperatures are included in Supporting Information; Figures S6–S11).

However, at 243 K (Figure 3), some new very weak peaks emerge (indicated by red arrows in Figure 3), which match the simulated powder XRD pattern of phase **C**. On cooling from 243 to 223 K, both phase **A** and phase **C** co-exist in the powder sample, as evident from the diagnostic peaks for phase **A** (cyan arrows in Figure 3) and phase **C** (red arrows in Figure 3). Within this temperature range, the relative intensity of the powder XRD pattern due to phase **A** decreases while the relative intensity of the powder XRD pattern due to phase **C** increases, indicating that some of the starting sample of phase **A** transforms gradually to phase **C** on cooling from 243 to 223 K. Although the transition from phase **A** to phase **C** is first observed in the powder XRD data at a higher temperature than the exotherm at *T*_1_ ≈ 229 K in the DSC data (Figure 2), it is reasonable to assign this exothermic event in the DSC data to the transition from phase **A** to phase **C** (consistent also with conclusions from our single-crystal XRD study), recognizing that different cooling schedules were used in the DSC and powder XRD studies (specifically, continuous cooling at constant rate in the DSC measurements and intermittent cooling in the powder XRD measurements, involving periods at fixed temperature while measuring the powder XRD data and cooling at constant rate between these periods). Furthermore, single-crystal XRD results (Section 2.3) show that two different crystals undergo the transformation from phase **A** to phase **C** at different temperatures on cooling (in one case at 230 K and in the other case above 230 K). Thus, our observation that the amount of phase **C** relative to phase **A** increases progressively over a range of temperatures on cooling in the powder XRD experiment is fully consistent with our conclusion from the single-crystal XRD data that the transition from phase **A** to phase **C** may be initiated at different temperatures for different crystals.

**Figure 4:**
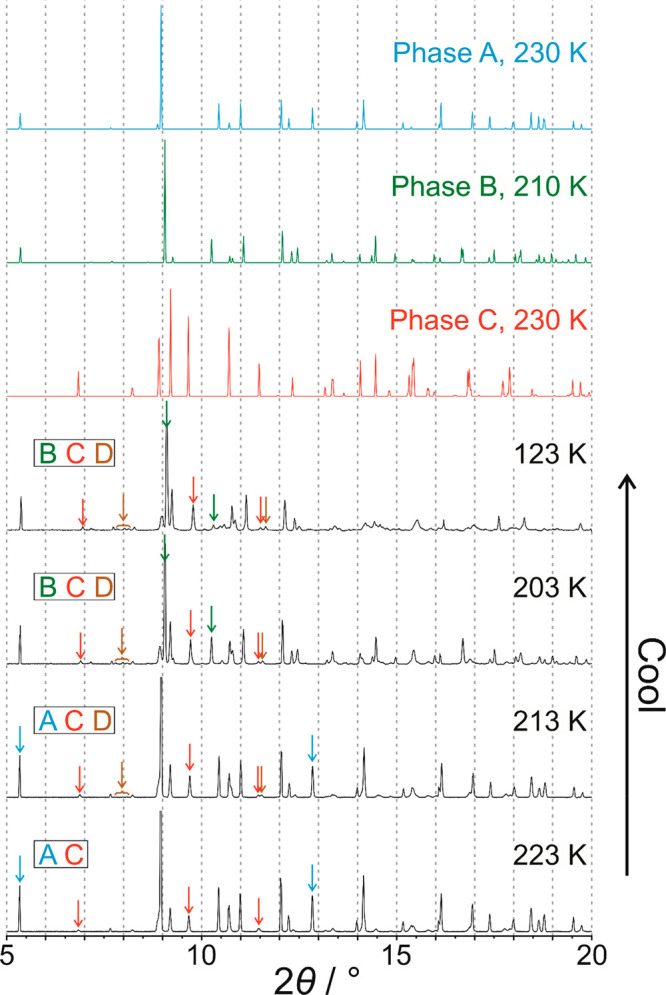
Powder XRD data recorded for 1-IA on cooling from 223 to 123 K. Peaks indicated by arrows are diagnostic peaks for phase **A** (cyan arrows), phase **B** (green arrows), phase **C** (red arrows), and phase **D** (brown arrows). Powder XRD patterns simulated from the crystal structures of phase **A** (cyan), phase **B** (green), and phase **C** (red) are also shown. The phases present at each temperature are indicated at the left side.

At 213 K (Figure 4), a new set of weak peaks appears in the powder XRD data (indicated by brown arrows in Figure 4) representing the emergence of another crystalline phase between 223 and 213 K. The new peak positions do not correspond to either of the low-temperature phases (**B** or **C**) observed in our single-crystal XRD studies and are assigned to another low-temperature phase of unknown structure (denoted phase **D**). The formation of phase **D** is assigned to the exothermic event labeled *T*_2_ in the cooling cycle of the DSC data (Figure 2).

**Figure 5:**
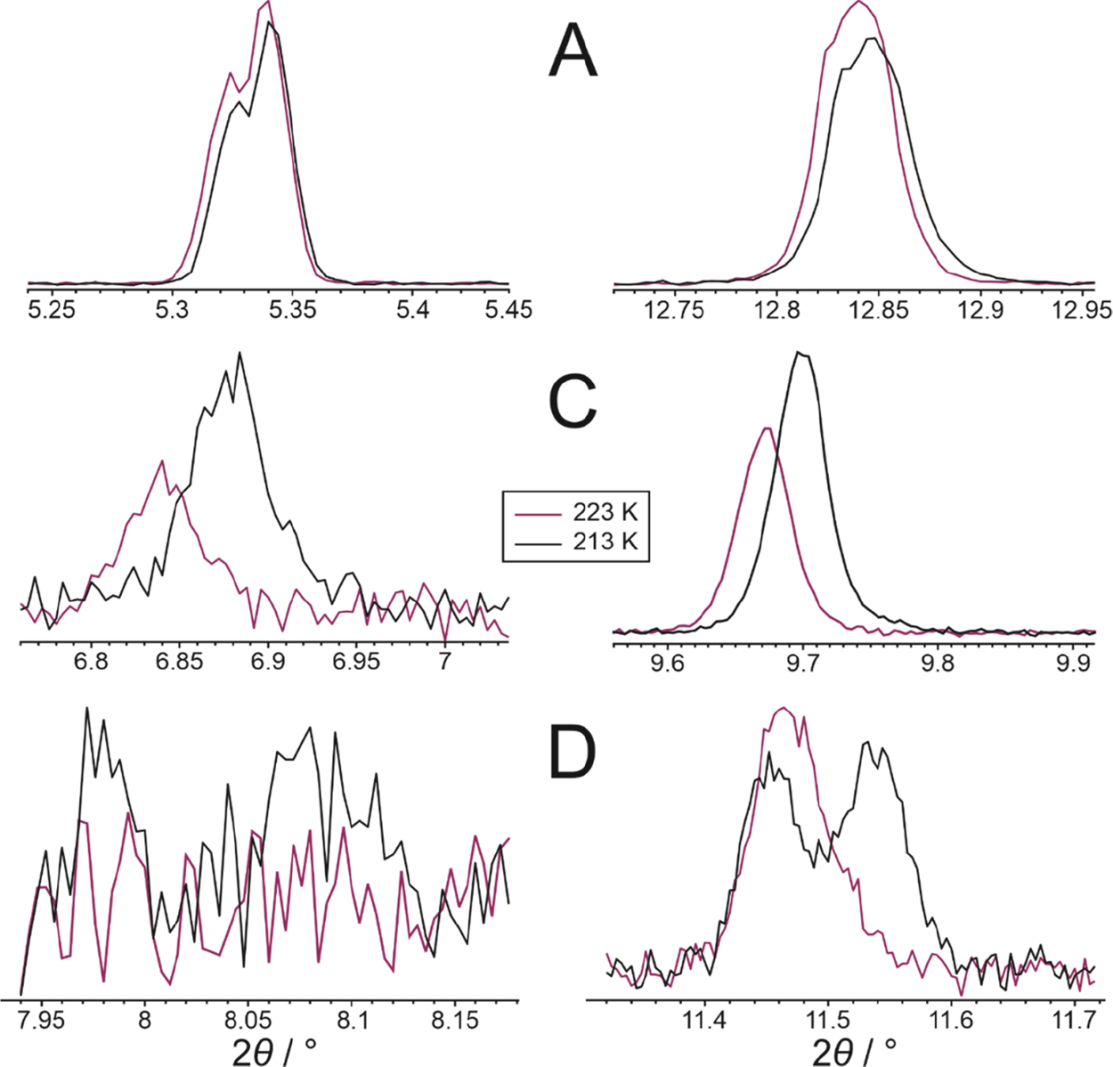
Diagnostic peaks for phases **A**, **C**, and **D** in the powder XRD data recorded for 1-IA at 223 K (purple) and 213 K (black) on cooling. The diagnostic peaks for phase **D** are at 2θ = 7.98°, 8.09°, and 11.54° (the peak at 2θ = 11.54° is overlapped with a peak at 2θ = 11.46° due to phase **C**).

To establish whether phase **D** is produced from phase **A** or from phase **C** on cooling, we consider the change in the relative intensities of the powder XRD patterns due to phases **A** and **C** between 223 and 213 K (Figure 5). It is clear from Figure 5 that the appearance of peaks due to phase **D** in the powder XRD data at 213 K is associated with a decrease in the relative intensity of the powder XRD pattern due to phase **A** at 213 K (relative to 223 K). In contrast, the relative intensity of the powder XRD pattern due to phase **C** at 213 K actually increases slightly (relative to 223 K). These observations suggest that, between 223 and 213 K on cooling, some amount of phase **A** transforms to phase **D**, while some amount of phase **A** continues to transform to phase **C** (as also observed on cooling between 243 and 223 K).

On further cooling from 213 to 203 K (Figure 4), the powder XRD pattern due to phase **A** disappears completely, while new peaks characteristic of phase **B** are observed at 203 K (it is clear from the powder XRD data at 203 K that the diagnostic peaks for phase **A** are absent, while the diagnostic peak for phase **B** is present). Based on this change in the powder XRD data between at 213 and 203 K on cooling, the exothermic event observed at *T*_3_*≈* 211 K in the DSC data (Figure 2) is assigned to a transformation of the remaining amount of phase **A** in the powder sample to phase **B**. This assignment is also consistent with our single-crystal XRD results as the two crystals exhibiting behavior (i) that were studied at 210 K (crystal **1** and crystal **2** in Table S1) were both in phase **B** at 210 K. Furthermore, as noted in Section 2.3, phase **A** was never observed below *T*_3_*≈* 211 K in any of our single-crystal XRD studies.

Following the complete disappearance of phase **A** between 213 and 203 K in our powder XRD study, the powder sample comprised a mixture of phases **B**, **C**, and **D**. No further phase transitions are evident from the powder XRD data on cooling to 123 K (Figure 4), and the powder sample remained a mixture of phases **B**, **C**, and **D** throughout this temperature range. We also emphasize that there was no evidence for any transformations *between* phases **B**, **C**, and **D** during the cooling cycle of the variable-temperature powder XRD study.

#### 2.4.3 Evolution of Solid Phases on Heating

After completing the cooling cycle at 123 K in the powder XRD study, the sample capillary was translated along the axis of the capillary in order to expose a fresh part of the powder sample to the X-ray beam. This action was taken to ensure that the powder XRD data recorded in the heating cycle involved a part of the powder sample that had not been exposed to the X-ray beam in the cooling cycle, based on our concern that prolonged exposure to the X-ray beam may cause beam damage to the sample (see Section 4.3). The powder XRD datasets recorded at 123 K before and after translating the capillary (Figure S12) show that the part of the powder sample exposed to the X-ray beam during the cooling cycle and the part of the powder sample *not* exposed to the X-ray beam during the cooling cycle both contained the same mixture of phases **B**, **C**, and **D**, although with some differences in the relative amounts of these phases (assessed from the relative intensities of the powder XRD patterns characteristic of each phase). Specifically, the part of the sample exposed to the X-ray beam during the cooling cycle contained a higher proportion of phase **B**, whereas the part of the sample that was not exposed to the X-ray beam during the cooling cycle contained a higher proportion of phase **C**. Phase **D** is also present in both parts of the sample, with a higher proportion in the part of the sample that was not exposed to the X-ray beam during the cooling cycle (Figure S12).

**Figure 6:**
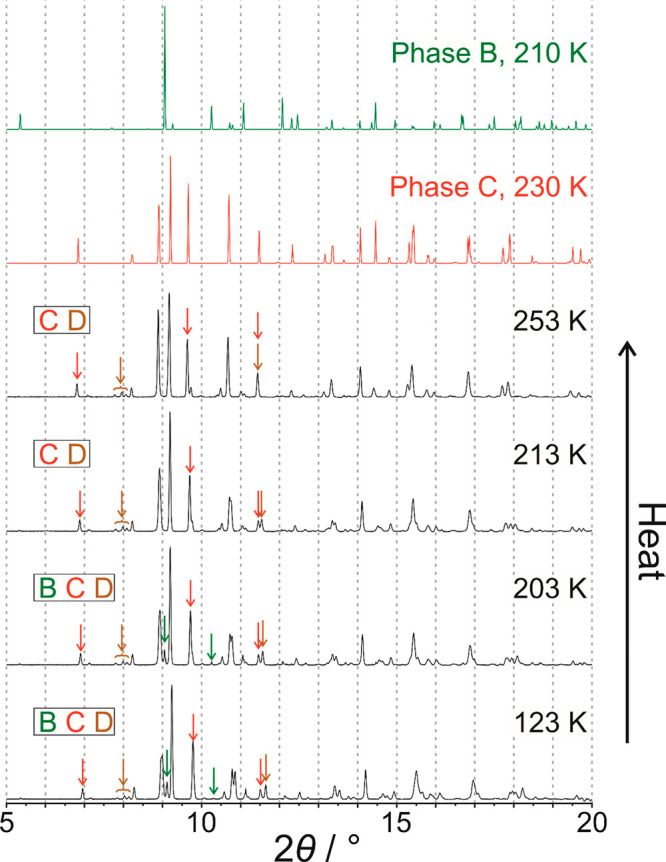
Powder XRD data recorded for 1-IA on heating from 123 to 253 K. Peaks indicated by arrows are diagnostic peaks for phase **B** (green arrows), phase **C** (red arrows), and phase **D** (brown arrows). Powder XRD patterns simulated from the crystal structures of phase **B** (green) and phase **C** (red) are also shown. The phases present at each temperature are indicated at the left side.

**Figure 7:**
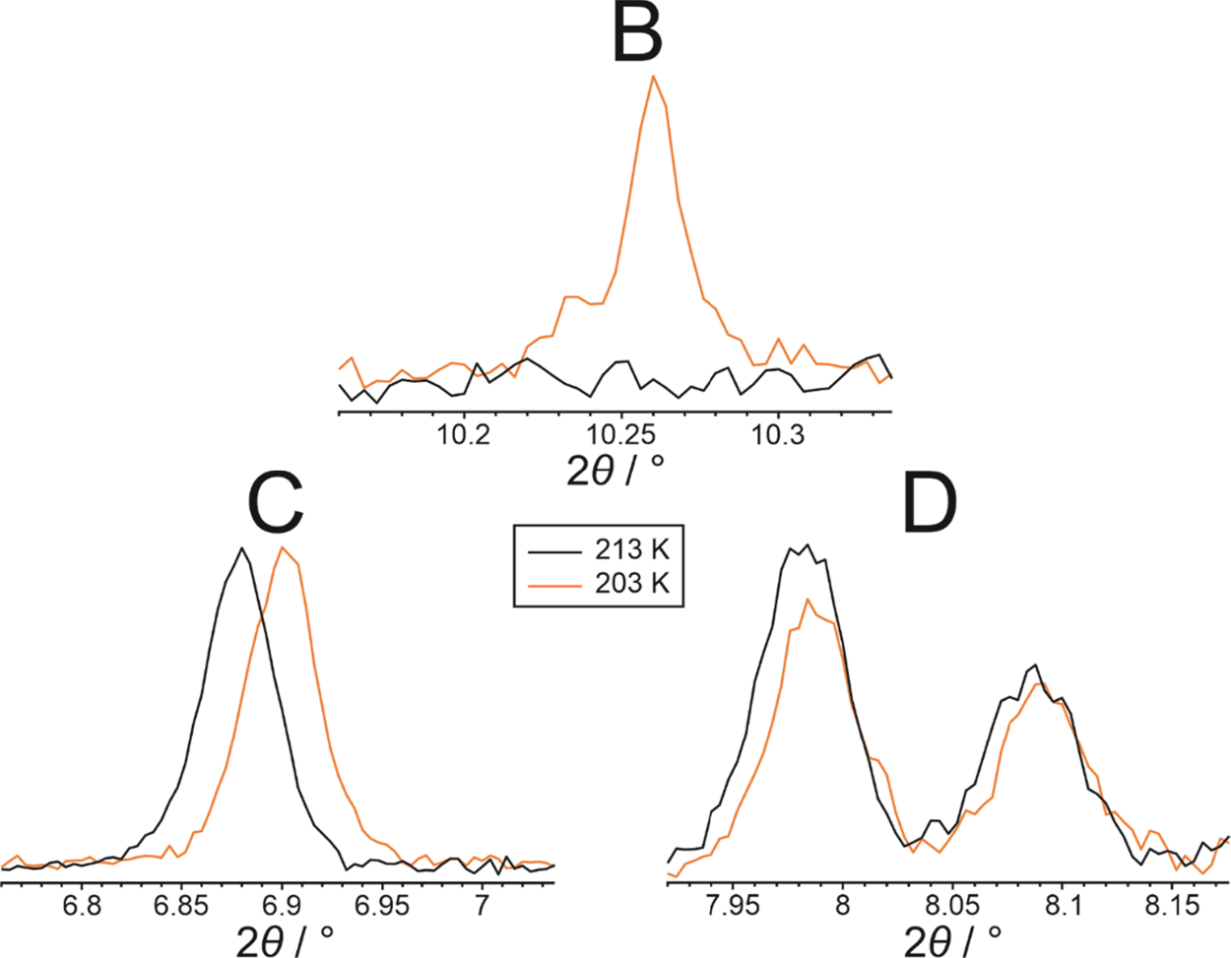
Diagnostic peaks for phases **B**, **C**, and **D** in the powder XRD data recorded for 1-IA at 203 K (orange) and 213 K (black) on heating.

On heating the powder sample from 123 to 203 K (Figure 6), no changes are observed in the powder XRD data, and the sample remains as a mixture of phases **B**, **C**, and **D**. However, the powder XRD data recorded at 213 K are consistent with a mixture of phases **C** and **D**, with the loss of the powder XRD pattern due to phase **B** and with no evidence for the appearance of any new crystalline phase. To assess whether the loss of phase **B** arises by transformation of phase **B** to phase **C** or by transformation of phase **B** to phase **D**, we consider the changes in the relative intensities of peaks in the powder XRD data between 203 and 213 K. As shown in Figure 7, on heating from 203 to 213 K, the intensity of the diagnostic peak for phase **B** decreases to zero, while the intensities of the diagnostic peaks for phase **C** remain constant and the intensities of the diagnostic peaks for phase **D** increase. From these observations, it is clear that phase **B** transforms to phase **D** on heating from 203 to 213 K. On this basis, the endotherm at *T*_4_ ≈ 211 K in the heating cycle of the DSC data (Figure 2) is assigned to a transformation from phase **B** to phase **D**.

**Figure 8:**
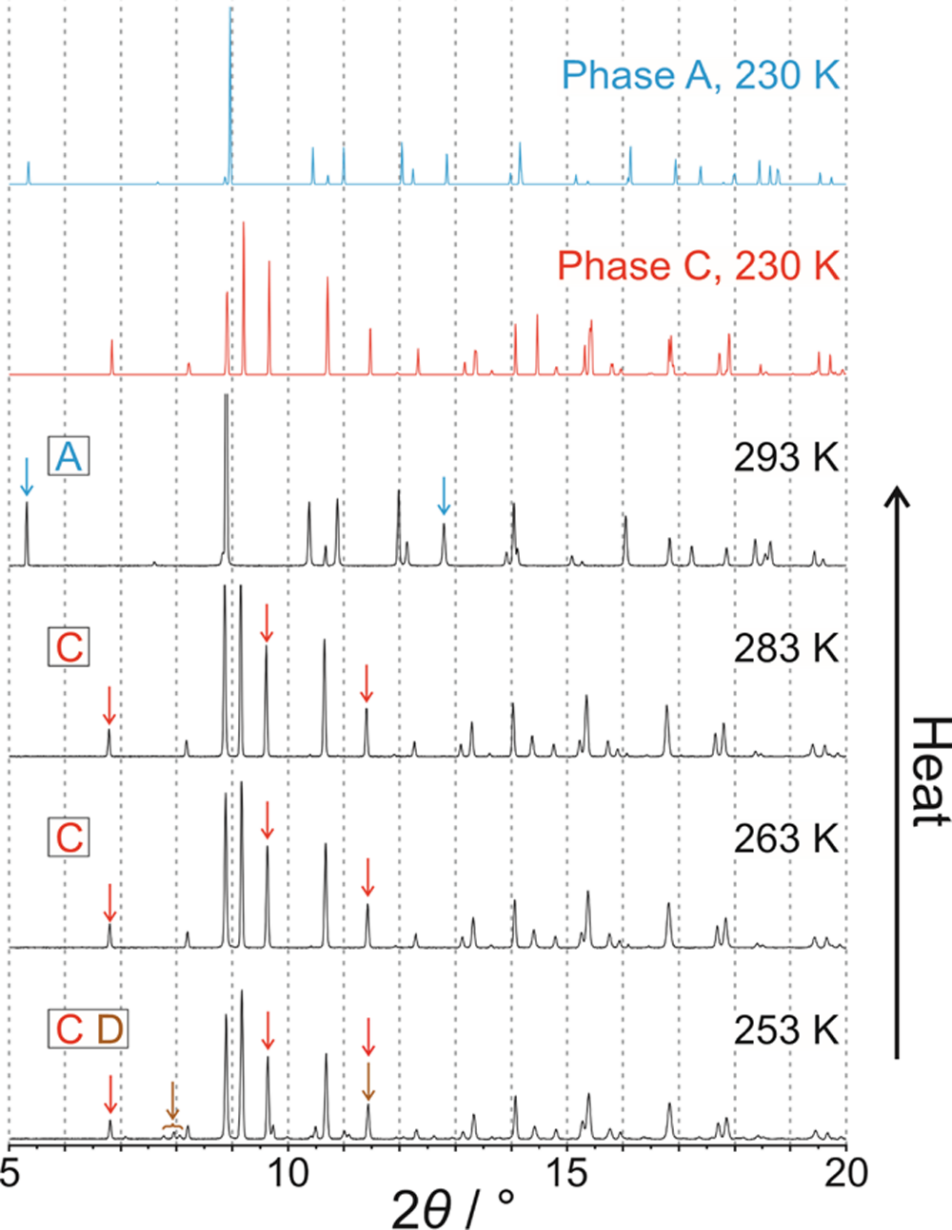
Powder XRD data recorded for 1-IA on heating from 253 to 293 K. Peaks indicated by arrows are diagnostic peaks for phase **A** (cyan arrows), phase **C** (red arrows), and phase **D** (brown arrows). Powder XRD patterns simulated from the crystal structures of phase **A** (cyan) and phase **C** (red) are also shown. The phases present at each temperature are indicated at the left side.

On further heating, no significant changes arise in the powder XRD data between 213 and 253 K (Figure 6), and the sample remains as a mixture of phases **C** and **D**. However, at 263 K, the powder XRD data (Figure 8) are consistent with a monophasic sample of phase **C**, indicating that phase **D** has transformed to phase **C** between 253 and 263 K. On this basis, the endotherm at *T_5_* ≈ 255 K in the heating cycle of the DSC data (Figure 2) is assigned to a transformation from phase **D** to phase **C**. No further changes are observed in the powder XRD data from 263 to 283 K, with the sample remaining as phase **C**, but the powder XRD data recorded at 293 K (Figure 8) indicate that the sample has transformed to a monophasic sample of phase **A**. On this basis, the endotherm at *T*_6_ ≈ 284 K in the heating cycle of the DSC data (Figure 2) is assigned to a transformation from phase **C** to phase **A**.

In a separate powder XRD experiment, carried out on a laboratory powder XRD setup, a monophasic sample of phase **A** was heated from ambient temperature (290 K) to a temperature (345 K) just below the melting temperature (348 K). The powder XRD data (Figure S13) confirm that the sample remains as phase **A** at all temperatures within the range investigated, with no evidence that phase **A** undergoes any phase transition on heating to the melting temperature, which is fully consistent with conclusions from DSC data (see Section 2.2 and Figure 2).

#### 2.4.4 Comment on the Sequence of Phase Transitions on Cooling and Heating

**Figure 9:**
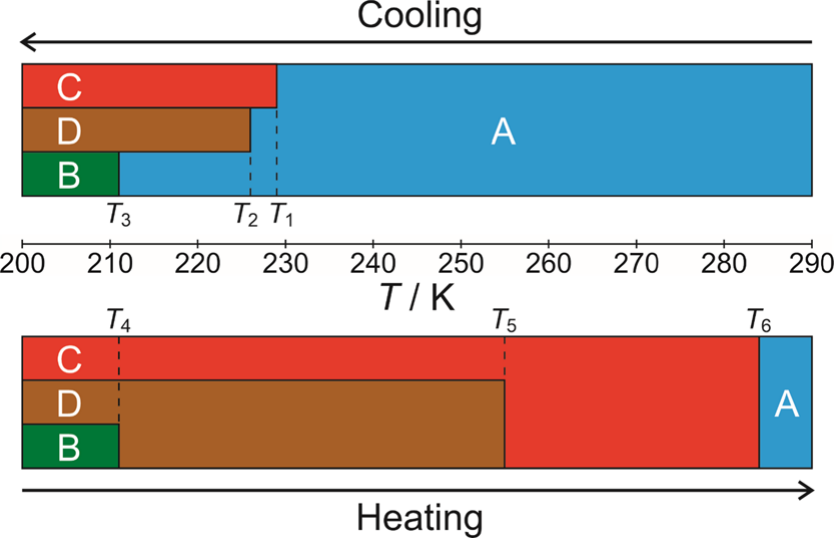
Summary of the sequence of phase transitions on cooling a powder sample of 1-IA and on subsequent heating. In the cooling cycle, different crystals in the initial powder sample of phase **A** undergo the three distinct transitions to phase **B**, phase **C**, and phase **D**. The temperature indicated for each phase transition is taken from the corresponding thermal event observed in DSC data (*T*_1_ = 229.1 K, *T*_2_ = 226.1 K, *T*_3_ = 210.7 K, *T*_4_ = 211.5 K, *T*_5_ = 255.1 K, *T*_6_ = 283.6 K).

From our variable-temperature powder XRD study, the specific phases of 1-IA that are involved in phase transitions on cooling from 293 to 123 K and on subsequent heating to the melting temperature (348 K) are summarized in Figure 9. As the powder XRD data were recorded in steps of 10 K (for reasons explained above), the specific temperatures at which the phase transitions occur in the powder XRD experiment cannot be established accurately. Nevertheless, there is a clear correlation between the temperature regions in which phase transitions are evident from the powder XRD data and the temperatures at which thermal events are observed in our DSC data (Figure 2), and it is reasonable to correlate the sequence of thermal events in the DSC data on cooling and heating with the sequence of structural phase transitions deduced from the powder XRD data on cooling and heating. On this basis, the summary in Figure 9 assigns the temperatures of the thermal events in the DSC data to specific structural phase transitions identified from our powder XRD results.

### 2.5 Structural Properties of Phases **A**, **B**, and **C**

#### 2.5.1 Structural Properties of Phase **A**

**Figure 10:**
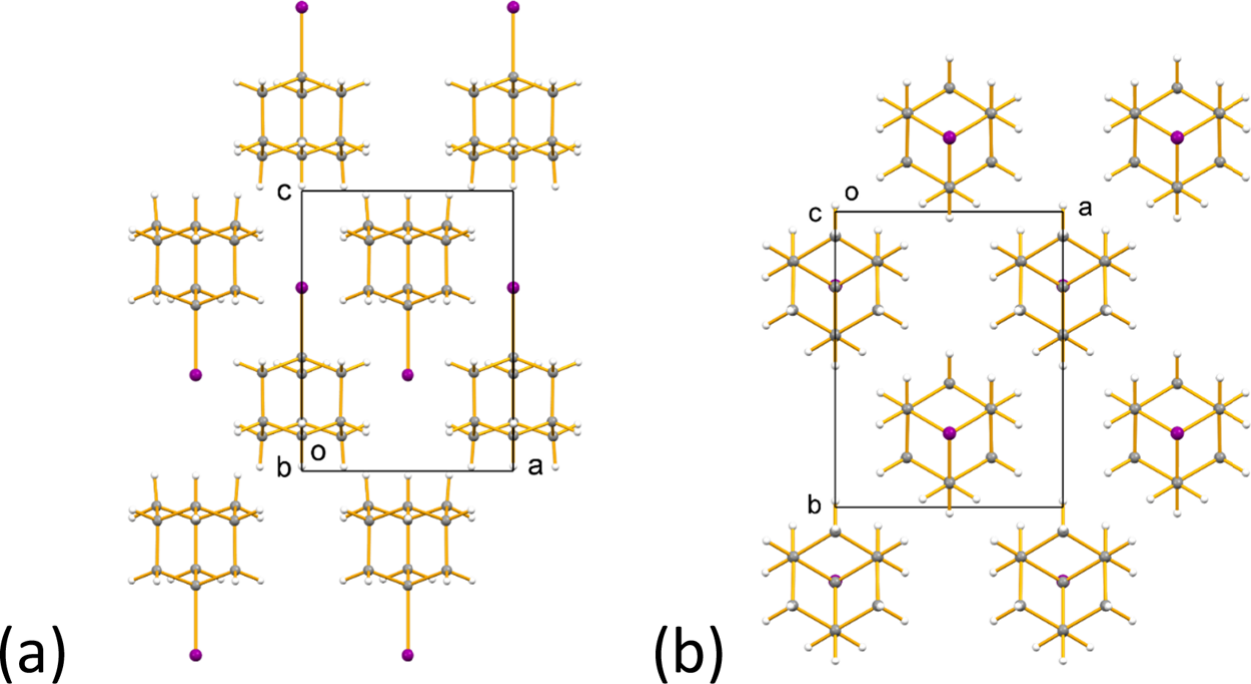
Crystal structure of phase **A** of 1-IA at 290 K viewed along (a) the *b*-axis and (b) the *c*-axis.

The crystal structure of phase **A** (orthorhombic, space group *Pm*2_1_*n*, *Z’* = 1/2) determined from single-crystal XRD data is shown in Figure 10 (we note that the structure of phase **A** is discussed in space group *Pm*2_1_*n*, rather than the conventional *Pmn*2_1_ setting for this space group, in order to facilitate subsequent comparison to the structure of phase **B**). The asymmetric unit comprises one half molecule of 1-IA, with each molecule located on a crystallographic mirror plane parallel to the *bc*-plane. The C–I bond vector lies in this mirror plane and is oriented parallel to the *c*-axis. The structure contains rows of 1-IA molecules along the *c*-axis. Within a given row, the C–I bond vectors of all molecules are oriented in the same direction (along the positive direction of the *c*-axis for some rows and along the negative direction of the *c*-axis for other rows). Relative to a given row in which the C–I bond vectors are oriented along the positive direction of the *c*-axis, the C–I bond vectors of all molecules in the four nearest neighbor rows (related to the reference row by vectors ±(***a*** ± ***b***)/2 in the projection shown in Figure 10b) are oriented along the negative direction of the *c*-axis.

We note that the crystal structure of phase **A** reported previously by Foulon and Gors^24^ is described by space group *Pmnn* and exhibits disorder of each 1-IA molecule between two orientations differing by 60° rotation about the C–I bond axis. In contrast, our analysis of single-crystal XRD data for phase **A** in this work indicates that the correct space group is *Pmn*2_1_, and the crystal structure does not exhibit disorder of the molecular orientation corresponding to 60° rotation about the C–I bond axis as reported by Foulon and Gors. This conclusion is further supported by difference Fourier electron density analysis (see Supporting Information, Section S2) which confirms that no significant residual electron density exists in the sites that would be occupied by carbon atoms of 1-IA molecules in the orientation corresponding to 60° rotation about the C–I bond axis.

#### 2.5.2 Dynamic Properties of Phase **A**

**Figure 11:**
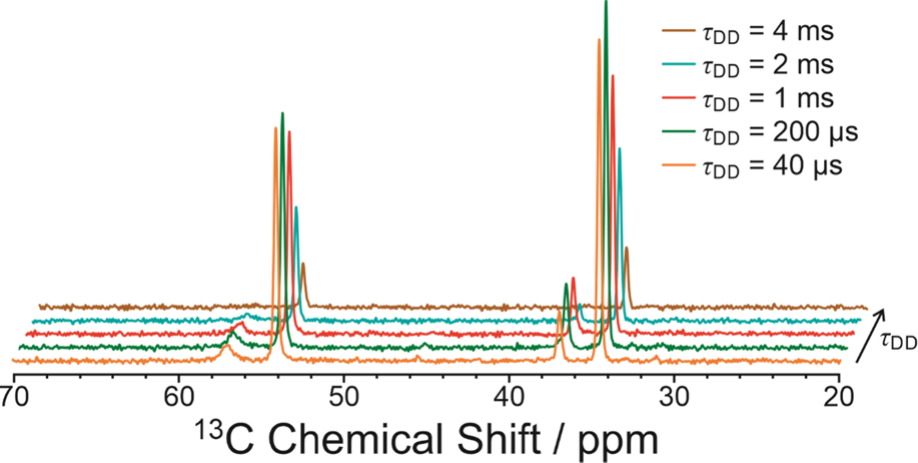
High-resolution solid-state ^13^C NMR spectra recorded for phase **A** of 1-IA at 293 K using the ^1^H → ^13^C cross-polarization technique with dipolar dephasing at different values of the dipolar dephasing delay (τ_DD_). The isotropic peaks are assigned to the following environments in the 1-IA molecule: 57.1 ppm [C(1)I], 54.3 ppm [C(2)H_2_], 36.9 ppm [C(3)H], and 34.5 ppm [C(4)H_2_].

To assess whether any significant motion of the 1-IA molecules occurs in phase **A**, high-resolution solid-state ^13^C NMR spectra (Figure 11) were recorded using the dipolar dephasing technique,^25,26^ which gives insights into the occurrence of dynamic processes in organic materials.^27−31^ Specifically, in dipolar dephasing solid-state ^13^C NMR spectra recorded with dipolar dephasing delay (τ_DD_) longer than about τ_DD_ ≈ 40 μs, isotropic peaks for ^13^C nuclei directly bonded to ^1^H have significant intensity *only* if the ^13^CH_*n*_ (*n* = 1, 2, 3) moiety is dynamic. In the dipolar dephasing solid-state ^13^C NMR spectra recorded for phase **A** at ambient temperature (Figure 11), the isotropic peaks at 54.3, 36.9, and 34.5 ppm (representing ^13^CH and ^13^CH_2_ environments in 1-IA) have significant intensity at τ_DD_ = 40 μs, and also at much longer values of τ_DD_ (e.g., τ_DD_ = 4 ms), clearly indicating that the 1-IA molecules are dynamic in phase **A**. Quantitative fitting based on Gaussian decay^26,28^ of the intensity of each isotropic peak as a function of τ_DD_ gives time constants (*T*_DD_) of 2.54, 1.81, and 2.63 ms for the peaks at 54.3, 36.9, and 34.5 ppm, respectively. Values of *T*_DD_ of this order of magnitude for ^13^CH and ^13^CH_2_ environments are indicative of significant reorientational dynamics.^31^

The crystal structure of phase **A** determined from single-crystal XRD data (Figure 10) represents a time-average over the dynamic process, and as this time-averaged crystal structure does not exhibit disorder, the dynamic process must involve 3-fold 120° jumps of each 1-IA molecule about the molecular 3-fold symmetry axis (parallel to the C–I bond). As the symmetry of the jump process matches a molecular symmetry element, the time-averaged crystal structure is indistinguishable from a static ordered structure.

#### 2.5.3 Structural Properties of Phase **B**

**Figure 12:**
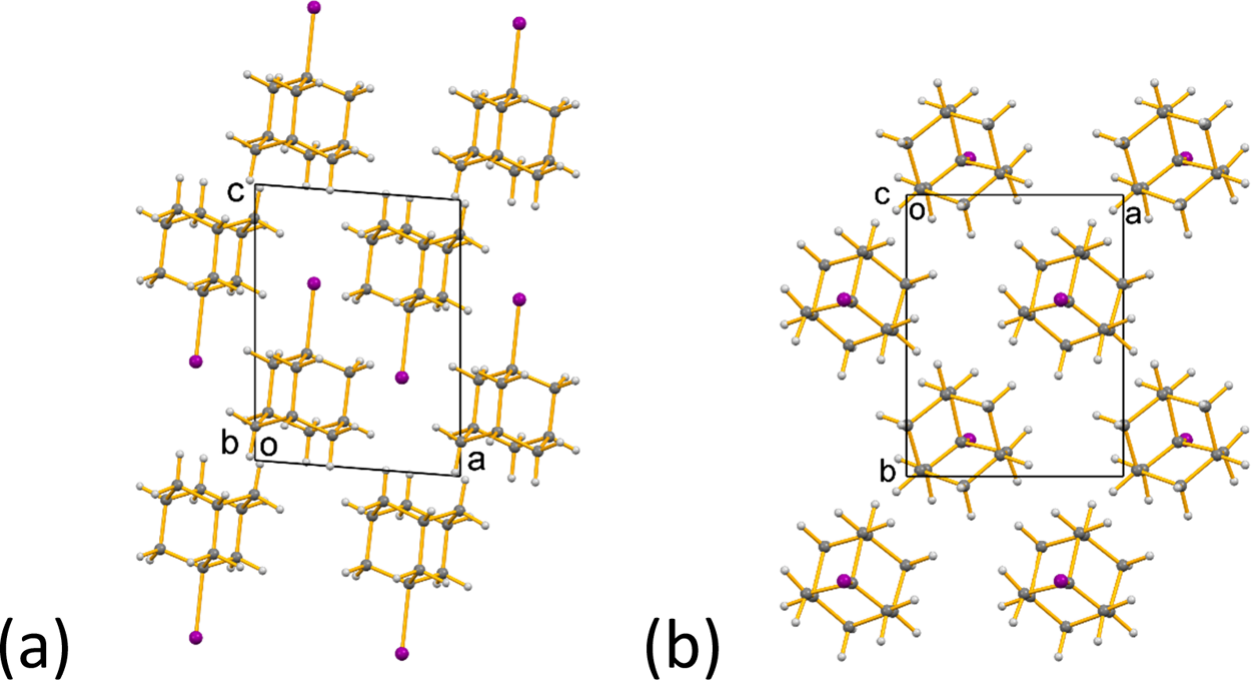
Crystal structure of phase **B** of 1-IA at 175 K viewed along (a) the *b*-axis and (b) the *c*-axis.

The crystal structure of phase **B** (monoclinic, space group *P*2_1_, *Z’* = 1), determined from single-crystal XRD data, is show in Figure 12. The structure of phase **B** is very similar to phase **A**, although with lower symmetry, and a group–subgroup relationship exists between these phases (phase **A**, *Pm*2_1_*n*; phase **B**, *P*2_1_). The structural similarity between phase **A** and phase **B** is clear from the overlay of these structures in Figure S14. The structure of phase **B** contains rows of molecules along the *c*-axis (similar to the rows of molecules along the *c*-axis in phase **A**), with adjacent molecules related by translation along this axis. Within a given row in phase **B**, the C–I bond axes of all molecules are aligned parallel to each other, with the C–I bond of each molecule tilted by 5.64° from the translation axis (*c*-axis) that defines the rows of molecules. In contrast, in phase **A**, the C–I bonds of all molecules are exactly parallel to the translation axis (*c*-axis). Also, while all molecules in phase **A** lie on a mirror plane parallel to the *bc*-plane, each molecule in phase **B** is rotated by ca. 11.5° around the C–I bond axis relative to the *bc*-plane.

#### 2.5.4 Structural Properties of Phase **C**

**Figure 13:**
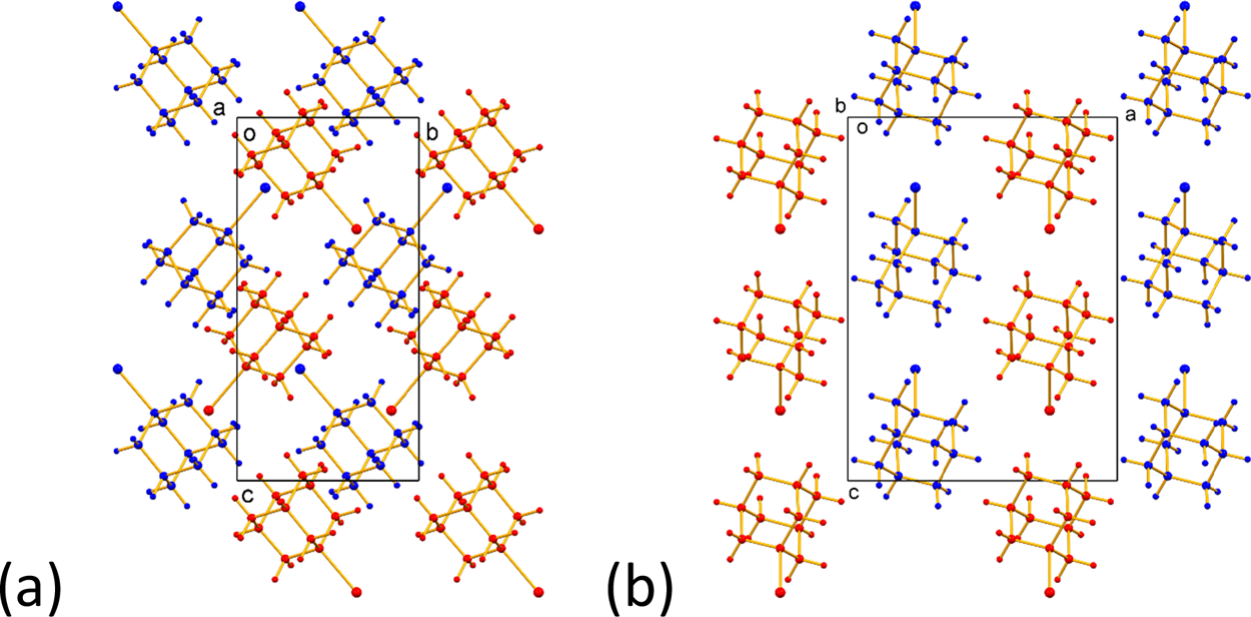
Crystal structure of phase **C** of 1-IA at 230 K (showing only one component of the disordered structure) viewed along (a) the *a*-axis and (b) the *b*-axis. For clarity, the molecules of 1-IA are shown as red or blue to distinguish adjacent layers of molecules parallel to the *bc*-plane. Each row of molecules along the *c*-axis discussed in the text contains molecules of the same color (either blue or red). The alternation of the orientations of the C–I bonds of adjacent molecules in each row along the *c*-axis is clearly seen in (a).

The crystal structure of phase **C** (monoclinic, space group *P*2_1_/c, *Z’* = 1) determined from single-crystal XRD data is shown in Figure 13 and exhibits several contrasting structural features compared to phases **A** and **B**. First, the 1-IA molecules in phase **C** are disordered between two orientations (Figure S15) that are related by 60° rotation about the C–I bond axis, with essentially equal occupancies of the two orientations (see Section 4.4 and Table S1). In contrast, no orientational disorder is observed in the time-averaged and space-averaged description of the crystal structures of phases **A** and **B** determined from single-crystal XRD data. For simplicity, we discuss the crystal structure of phase **C** (Figure 13) in terms of one component of the disordered structure. In this structure, the molecules are arranged in rows parallel to the *c*-axis, with the C–I bond of each molecule tilted by 40.3° from the *c*-axis and lying essentially parallel to the *bc*-plane. For adjacent molecules along the row, the direction of tilt alternates between +40.3° (i.e., C–I bond tilted from the *c*-axis toward the positive direction along the *b*-axis) and – 40.3° (i.e., C–I bond tilted from the *c*-axis toward the negative direction along the *b*-axis). In contrast, the C–I bonds of the molecules within each row in phase **A** are strictly parallel to the translation axis (*c*-axis), and the C–I bonds of the molecules within each row in phase **B** are tilted by only 5.64° from the translation axis (*c*-axis).

We note that phase **C** is isostructural with the low-temperature phase of 1-bromoadamantane^15^ and the low-temperature ordered phase III of 1-chloroadamantane.^10^ However, in these structures, orientational disorder around the C–X bond axis is not observed, in contrast to the situation reported here for phase **C** of 1-IA.

#### 2.5.5 Structural Relationships between Phases A, B, and C

The crystal structures of phase **A** (Figure 10) and phase **B** (Figure 12) share several common features, as highlighted in the discussion in Section 2.5.3, whereas the transformation from phase **A** to phase **C** involves substantially greater structural reorganization and changes in molecular orientations. In particular, molecules related by translation along a row in phase **A** (parallel to the *c*-axis) undergo a rotation by +40.3° or –40.3° about the *a*-axis (the sign of rotation alternates for adjacent molecules along the row) in transforming to phase **C**.

The discontinuity in unit cell volume associated with the transition from phase **A** to phase **B** (Figure S3; Table S1) corresponds to a change in the volume per molecule (*V*_m_) of Δ*V*_m_ ≈ −3.5 Å^3^ (determined from data for crystal **2** in Table S1: phase **A**, *V*_m_ = 252.9 Å^3^ at 215 K; phase **B**, *V*_m_ = 249.4 Å^3^ at 210 K). Thus, phase **B** has higher density than phase **A** at a comparable temperature. The phase transition from phase **A** to phase **C** is associated with a larger discontinuity in unit cell volume (Figure S5; Table S1), corresponding to a change in the volume per molecule of Δ*V*_m_ ≈ −8.4 Å^3^ (determined from data for crystal **8** in Table S1: phase **A**, *V*_m_ = 254.6 Å^3^ at 230 K; phase **C**, *V*_m_ = 246.2 Å^3^ at 230 K) and indicating that phase **C** has higher density than phase **A** at the same temperature.

The densities of phases **B** and **C** can also be compared at a temperature (175 K) for which single-crystal XRD data were recorded for both phases. As no individual crystal in the single-crystal XRD study existed in both phase **B** and phase **C**, the volume per molecule in each phase is taken as the average over all crystals for which the unit cell volume was measured at 175 K for phase **B** (*V*_m_ = 246.9 Å^3^; average value for crystals **1**, **3**, and **5** in Table S1) and as the average over all crystals for which the unit cell volume was measured at 175 K for phase **C** (*V*_m_ = 240.3 Å^3^; average value for crystals **7** and **8** in Table S1). Clearly, phase **C** has higher density than phase **B** at this temperature.

## 3 Concluding Remarks

We have shown through the application of a multi-technique strategy that 1-IA exhibits complex phase transition behavior on cooling below ambient temperature and on subsequent heating to the melting temperature, as summarized in Figure 9. Our single-crystal XRD results indicate that individual crystals of phase **A** undergo only one phase transition on cooling. Among the crystals studied by single-crystal XRD, the transition from phase **A** to phase **B** was observed for some crystals and the transition from phase **A** to phase **C** was observed for other crystals, while the transition from phase **A** to phase **D** was not observed for any crystals. The results from our variable-temperature powder XRD study are fully consistent with the observations from single-crystal XRD. Thus, our powder XRD data show (in conjunction with DSC data to assign accurate transition temperatures) that, on cooling a monophasic powder sample of phase **A** from ambient temperature, some of the powder sample of phase **A** transforms to phase **C** at *T*_1_ ≈ 229 K, then some of the remaining phase **A** in the powder sample transforms to phase **D** at *T*_2_ ≈ 226 K, and finally, all of the remaining amount of phase **A** in the powder sample transforms to phase **B** at *T*_3_ ≈ 211 K, resulting in a triphasic mixture of phases **B**, **C**, and **D** below this temperature. During the cooling process, each of the phases **B**, **C**, and **D** is formed *directly* from phase **A**, and there is no evidence for the occurrence of any transformations *between* phases **B**, **C**, and **D** on cooling.

We note that previous studies of 1-IA at low temperature, using thermal analysis^23^ and solid-state NMR^22^ techniques, reported a phase transition at ca. 211 K on cooling, which is consistent with the phase transition from phase **A** to phase **B** observed in the present work. However, these previous studies did not report any evidence for the other low-temperature phase transitions reported here and did not report structural properties of any low-temperature phases.

On subsequently heating the mixture of phases **B**, **C**, and **D**, our powder XRD and DSC studies show that phase **B** transforms to phase **D** at *T*_4_ ≈ 211 K (resulting in a mixture of phases **C** and **D**), and then phase **D** transforms to phase **C** at *T*_5_ ≈ 255 K, resulting in a monophasic sample of phase **C**. Finally, on further heating, phase **C** transforms to phase **A** at *T*_6_ ≈ 284 K. Phase **A** does not undergo any further phase transitions until melting at *T*_melt_ ≈ 348 K.

The crystal structure of phase **D** has not been determined in the present work as this phase was observed only as a relatively minor component in mixtures of phases in our low-temperature powder XRD study. The fact that phase **D** was obtained only as a relatively minor component suggests that the transformation from phase **A** to phase **D** may be a low-probability transformation pathway on cooling phase **A**, which would be consistent with the fact that phase **D** was not observed to be formed for any of the crystals studied in our low-temperature single-crystal XRD experiments. Clearly, a priority for future research is to establish a procedure to determine the crystal structure of phase **D**, including the opportunity to measure three-dimensional electron diffraction^32−39^ (3D-ED) data for individual crystallites within the mixture of phases of 1-IA (i.e., phases **B**, **C**, and **D**) that is produced on cooling a sample of phase **A** from ambient temperature to sufficiently low temperature (e.g., 123 K), followed by structure determination from any 3D-ED data recorded for crystallites of phase **D** within the mixture of phases.

Clearly, the phase transition behavior of 1-IA is complex, particularly given the unusual observation that different crystals of phase **A** can undergo different phase transformation pathways on cooling, either as individual single crystals or as crystallites within a polycrystalline powder sample. As all single crystals studied by single-crystal XRD were ostensibly identical, at least at the level of information revealed by XRD, and as all crystallites in the *monophasic* powder sample of phase **A** used in the powder XRD study are implicitly assumed to be identical, it is clear that different crystals/crystallites of phase **A** must actually differ from each other in other aspects (such as crystalline domain sizes, surface properties, and/or defects) that may significantly influence their low-temperature phase transition behavior. Clearly, other experimental strategies are required in order to elucidate the reasons underlying the observation that different crystals/crystallites of phase **A** follow different phase transition pathways on cooling, and such studies will be the focus of our future research.

Finally, we note that the diverse range of solid thiourea inclusion compounds^40−43^ that have been reported include cases containing adamantane^44,45^ and certain derivatives of adamantane as guest molecules within the one-dimensional tunnels of the thiourea host structure. In particular, the thiourea inclusion compound containing 1-bromoadamantane guest molecules has received attention as a linear dichroic filter material for X-ray polarization analysis,^46^ with potential applications in X-ray astronomy,^47^ and also in studies of X-ray birefringence.^48,49^ As it is well established that thiourea inclusion compounds containing other types of guest molecules exhibit interesting phase transition behavior,^50−57^ we anticipate that studies of phase transitions in thiourea inclusion compounds containing 1-halogenoadamantane guest molecules may also be an interesting avenue for future investigation.

## 4 Experimental Methods

### 4.1 Materials Preparation

Crystallization of 1-IA was carried out by slow evaporation of solvent from a solution of 1-IA in ethanol. The solution was placed in a vial and covered with a parafilm, which was punctured with a few pin holes to allow evaporation. This preparation method produced single crystals of suitable size and quality for single-crystal XRD studies. Powder XRD data recorded at ambient temperature for the crystallized material matched the simulated powder XRD data for phase **A**.

### 4.2 Differential Scanning Calorimetry

DSC data were recorded for a powder sample of 1-IA (ca. 5–10 mg) in a hermetically sealed aluminum pan using a TA Instruments Q2000 differential scanning calorimeter. Starting from ambient temperature, the sample was cooled to 190 K and then heated to 360 K. DSC data were recorded for cooling/heating rates of 5 K min^–1^ and for cooling/heating rates of 20 K min^–1^. Each DSC experiment was repeated. Calibrations for cell constant and enthalpy were performed with indium (*T*_m_ = 156.6 °C, Δ*H*_f_ = 28.71 J/g) according to the manufacturer’s instructions. Nitrogen was used as a purge gas at a flow rate of 50 mL/min for all the experiments.

### 4.3 Powder XRD

To confirm the identity of the crystallized material, powder XRD data were recorded at ambient temperature on a Bruker D8 instrument operating in transmission mode using CuKα_1_ radiation (Ge-monochromator). The polycrystalline sample was contained in three sealed glass capillaries, which were attached to the disc sample holder of the powder XRD instrument (2θ range, 4°–50°; step size, 0.017°; data collection time, 25 min).

Synchrotron powder XRD studies of the temperature-dependent structural properties of 1-IA were carried out on beamline I11 at Diamond Light Source^58^ for a powder sample of 1-IA packed in a borosilicate glass capillary (0.7 mm). Powder XRD data were recorded using the Position Sensitive Detector (PSD) on beamline I11 (λ = 0.82462 Å; step size, 0.004°; 2θ range, 1° to 92°; data collection time, ca. 1 s), with rotation of the sample around the capillary axis. As the material was considered to be potentially sensitive to beam damage, attenuation was applied (aluminum plate, 1 mm thickness) to the incident X-ray beam. The sample temperature was controlled using an Oxford Cryostream Plus, with the gas stream co-axial with the sample capillary. Powder XRD data were recorded at 293 K and at increments of 10 K on cooling to 123 K (cooling rate, 6 K min^–1^). We note that relatively large temperature increments of 10 K were used in order to limit the amount of exposure of the sample to the X-ray beam during the complete cooling cycle and hence to minimize the risk of beam damage. After cooling the sample to 123 K and recognizing that some beam damage may have occurred (observed by a brown coloration in the region of the powder sample exposed to the X-ray beam), the capillary was translated by ca. 4 mm toward the Cryostream, allowing a fresh part of the powder sample to be exposed to the X-ray beam. Powder XRD data were then recorded at increments of 10 K on heating from 123 to 293 K (heating rate, 6 K min^–1^).

High-temperature powder XRD data were recorded on an Agilent SuperNova Dual Atlas X-ray diffractometer with a mirror monochromator using CuKα radiation (λ = 1.5418 Å). The instrument was configured with the two-dimensional detector perpendicular to the incident X-ray beam direction, with a sample-to-detector distance of 100 mm. The powder sample was contained in a glass capillary (diameter, 6 mm) with the capillary axis aligned perpendicular to the incident X-ray beam direction; the sample was rotated around the capillary axis during data collection. Data were recorded for a monophasic powder sample of phase **A** of 1-IA from 290 to 345 K in increments of 5 K. Between each measurement temperature, the sample was heated at a rate of 6 K min^–1^. At each measurement temperature, an equilibration time of 1 min was allowed before recording the powder XRD data, with a data collection time of 2 min. The two-dimensional powder XRD data recorded by this method were converted to a conventional one-dimensional powder XRD dataset (intensity versus 2θ) by integration of the Debye–Scherrer rings in the two-dimensional images.

### 4.4 Single-Crystal XRD

Single-crystal XRD data were recorded as a function of temperature for several crystals of 1-IA on an Agilent SuperNova Dual Atlas diffractometer with a mirror monochromator using either CuKα (λ = 1.5418 Å) or MoKα (λ = 0.7107 Å) radiation. The temperature of the crystal was controlled using an Oxford Cryosystems cooling apparatus. Single crystals were selected by optical microscopy prior to recording the single-crystal XRD data. A typical experiment involved data collections at several temperatures on cooling, with each data collection carried out at a fixed temperature. The cooling rate between the temperatures of the data collections was 6 K min^–1^. Data collection times ranged from 10 to 60 min. In total, eight separate single-crystal XRD experiments were carried out involving measurement of data at several temperatures to probe the structural properties as a function of temperature on cooling. In each experiment, a new single crystal was used, and single-crystal XRD data were measured at selected temperatures on cooling within the range 290–120 K, as detailed in Table S1 (the temperature range was not necessarily the same for each crystal studied).

Structure solution and refinement from single-crystal XRD data were carried out using SHELXS^59^ and SHELXL.^60^ Refinement of non-hydrogen atoms was carried out using anisotropic atomic displacement parameters. Hydrogen atoms were inserted in idealized positions, and a riding model was used with *U*_iso_ for each hydrogen atom set at 1.2 times the value of *U*_eq_ for the carbon atom to which it is bonded. In the refinements, it was generally necessary to apply restraints on molecular geometry and atomic displacement parameters in order to limit the prolate elongation associated with librational/rotational freedom around the molecular 3-fold symmetry axis (parallel to the C–I bond). All crystals that underwent the transition from phase **A** (orthorhombic) to phase **B** (monoclinic) were twinned in phase **B**, with twin components related by 180° rotation around the *c*-axis. For phase **C**, the 1-IA molecule is disordered between two orientations related by 60° rotation around the molecular 3-fold symmetry axis (see Section 2.5.4), with essentially equal occupancies of the two orientations. For the specific structure determination of phase **C** shown in Table 1, the refined occupancies were 0.497(9) and 0.503(9). Relevant data for other crystals of phase **C** are included in Table S1.

Representative crystal structures of phase **A** (at several temperatures from 290 to 215 K), phase **B** (at 210 and 175 K) and phase **C** (at 230 K) determined in this work have been deposited in the Cambridge Structural Database (CSD reference numbers: 2221485–2221495). The CSD reference numbers of the specific structure determinations for which data are given in Table 1 are 2221489 (phase **A**), 2221494 (phase **B**), and 2221487 (phase **C**). For phase **A**, the crystal structures deposited in the CSD have the conventional setting of the *Pmn*2_1_ space group (as explained in Section 2.5.1, the discussion of phase **A** throughout the paper has used the *Pm*2_1_*n* setting of this space group).

### 4.5 Solid-State NMR

High-resolution solid-state ^13^C NMR data were recorded for a powder sample of 1-IA at 293 K on a Bruker AVANCE III spectrometer (20.0 T) at the U. K. High-Field (850 MHz) Solid-State NMR Facility (^13^C Larmor frequency, 213.81 MHz; ^1^H Larmor frequency, 850.23 MHz) with a 4 mm HXY MAS probe in double resonance mode (MAS frequency, 12 kHz) using ramped ^1^H → ^13^C cross-polarization (CP contact time, 2.0 ms). High-resolution solid-state ^13^C NMR spectra were recorded at 293 K (MAS frequency, 12 kHz) using two different pulse sequences: (i) ramp-CP^61,62^ and (ii) ramp-CP followed by dipolar dephasing.^25^ In pulse sequence (ii), the dipolar dephasing delay (τ_DD_) forms part of a rotor-synchronized echo before the start of signal acquisition.

## Data Availability

Additional supporting experimental data for this article may be accessed at 10.17035/d.2023.0249272501.

## Supporting Information Available

Additional DSC data; powder XRD data recorded at all temperatures in the variable-temperature synchrotron powder XRD study; powder XRD data recorded on heating phase **A** of 1-IA from 290 to 345 K; single-crystal XRD image showing the co-existence of phase **A** and phase **C** in a single crystal of 1-IA at 230 K; unit cell volume per molecule as a function of temperature for a crystal of phase **A** that transformed to phase **B** on cooling; unit cell volume per molecule as a function of temperature for a crystal of phase **A** that transformed to phase **C** on cooling; comparison of the crystal structures of phase **A** and phase **B**; orientational disorder in the crystal structure of phase **C**; crystallographic data from all eight single-crystal XRD studies to investigate changes in the structural properties of 1-IA as a function of temperature on cooling, starting from phase **A**; and results and discussion of difference Fourier analysis of the crystal structure of phase **A** (PDF)
