# Supplementary material for: Unraveling the Complex Solid-State Phase Transition Behavior of 1-Iodoadamantane, a Material for Which Ostensibly Identical Crystals Undergo Different Transformation Pathways
Source: Cryst Growth Des. 2023 Apr 12;23(5):3820–33. doi: 10.1021/acs.cgd.3c00223 (PMC10161194; doi:10.1021/acs.cgd.3c00223)
Supplement: Supplementary file 1 — cg3c00223_si_001.pdf [file cg3c00223_si_001.pdf]

Supporting Information

Unraveling the complex solid-state phase transition behavior of 1-iodoadamantane, a material for which ostensibly identical crystals undergo different transformation pathways

Okba Al Rahal,[1] Benson M. Kariuki,[1] Colan E. Hughes,[1] P. Andrew Williams,[1] Xiaoyan Xu,[2] Simon Gaisford,[2] Dinu Iuga,[3] Kenneth D. M. Harris*[1]

1 School of Chemistry, Cardiff University, Park Place, Cardiff CF10 3AT, Wales, U. K.

2 Department of Pharmaceutics, School of Pharmacy, University College London, 29-39 Brunswick Square, London WC1N 1AX, England, U. K.

3 Department of Physics, University of Warwick, Coventry, CV4 7AL, England, U. K.

* Author for correspondence: HarrisKDM@cardiff.ac.uk

<u>Section S1:</u> Additional Figures S1 – S14 and Table S1

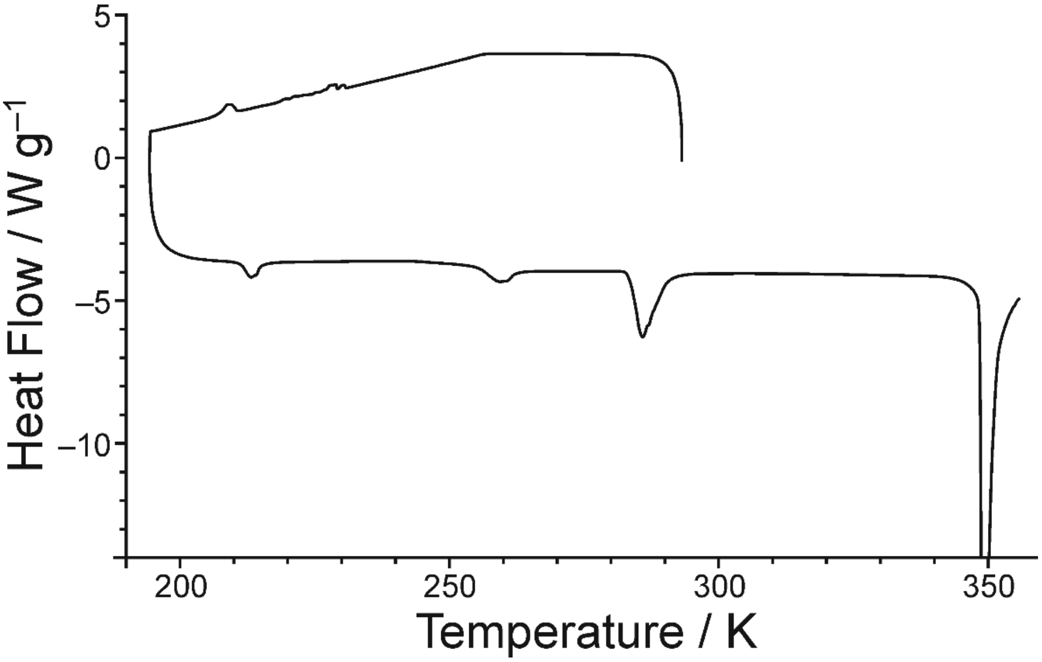

Figure S1. DSC data recorded for 1-IA with heating and cooling rates of 20 K min^{-1}, using the same batch of sample used to record Figure 2 in the main paper.

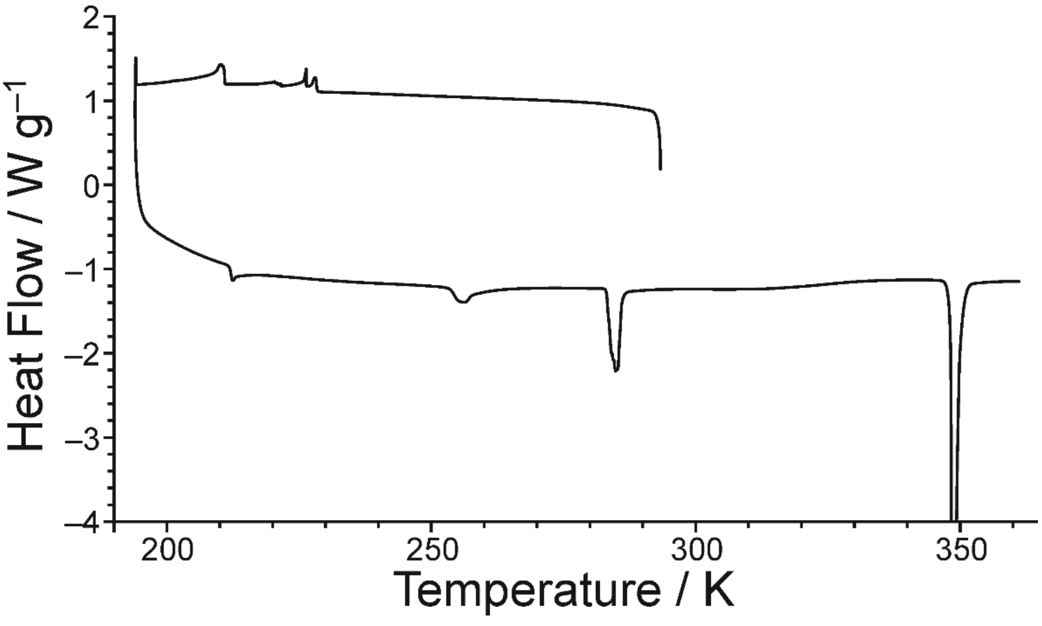

Figure S2. DSC data recorded for 1-IA with heating and cooling rates of 5 K min^{-1}, using a different batch of sample from that used to record Figure 2 in the main paper.

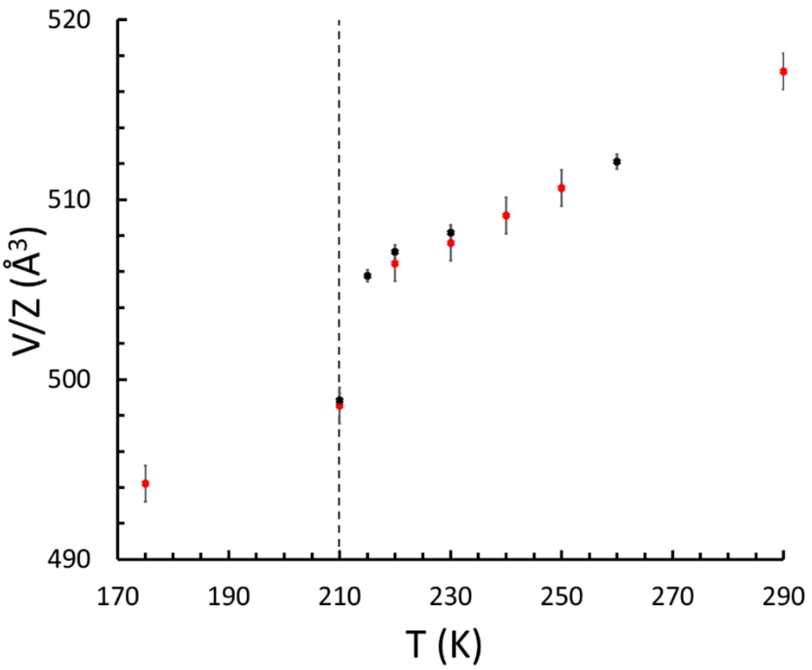

Figure S3. Unit cell volume per molecule as a function of temperature, determined from single-crystal XRD data, for two crystals of phase **A** that transformed to phase **B** on cooling, specifically crystal **1** (red data points) and crystal **2** (black data points) in Table S1. The dashed line shows the phase transition temperature established from DSC data.

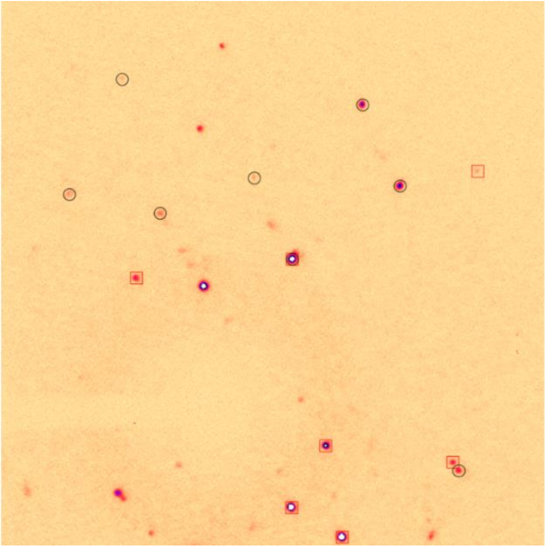

Figure S4. Single-crystal XRD image showing the simultaneous presence of diffraction peaks for phase **A** and phase **C** during the transformation of a single crystal of 1-IA from phase **A** to phase **C** at 230 K. Representative reflections from phase **A** and phase **C** are indicated by black circles and red squares, respectively, assigned on the basis of indexing the two independent XRD patterns (data for crystal **8** in Table S1).

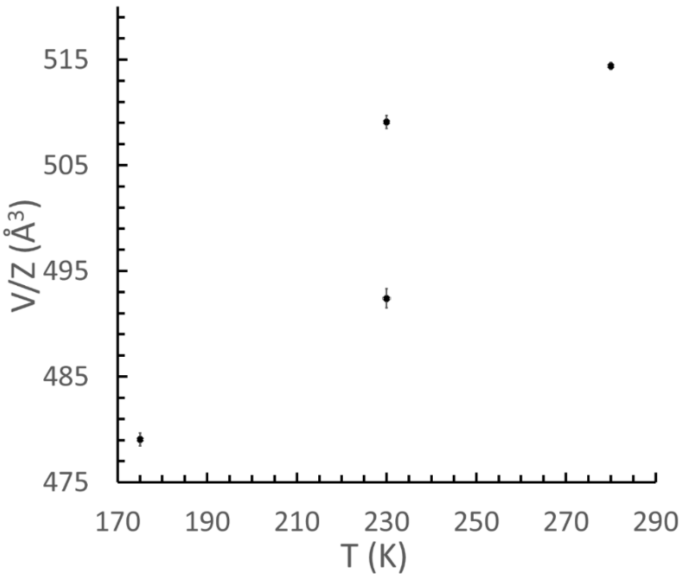

Figure S5. Unit cell volume per molecule as a function of temperature, determined from single-crystal XRD data, for a crystal of phase **A** that transformed to phase **C** on cooling (specifically, crystal **8** in Table S1). Data were recorded at 280 K, 230 K and 175 K. The crystal transformed from phase **A** to phase **C** while the temperature was held at 230 K, as discussed in the main paper.

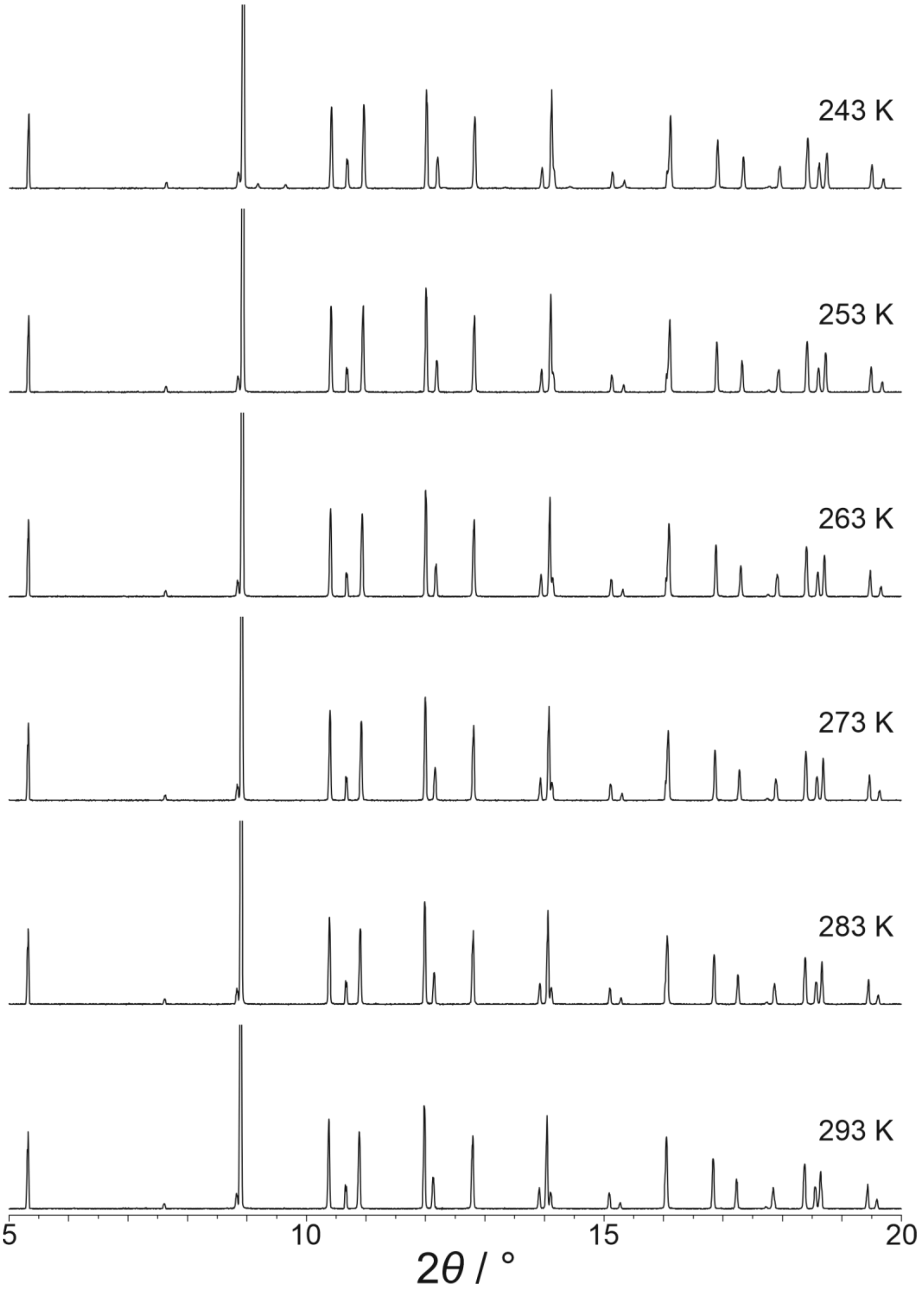

Figure S6: Powder XRD data recorded for 1-IA on cooling from 293 K to 243 K.

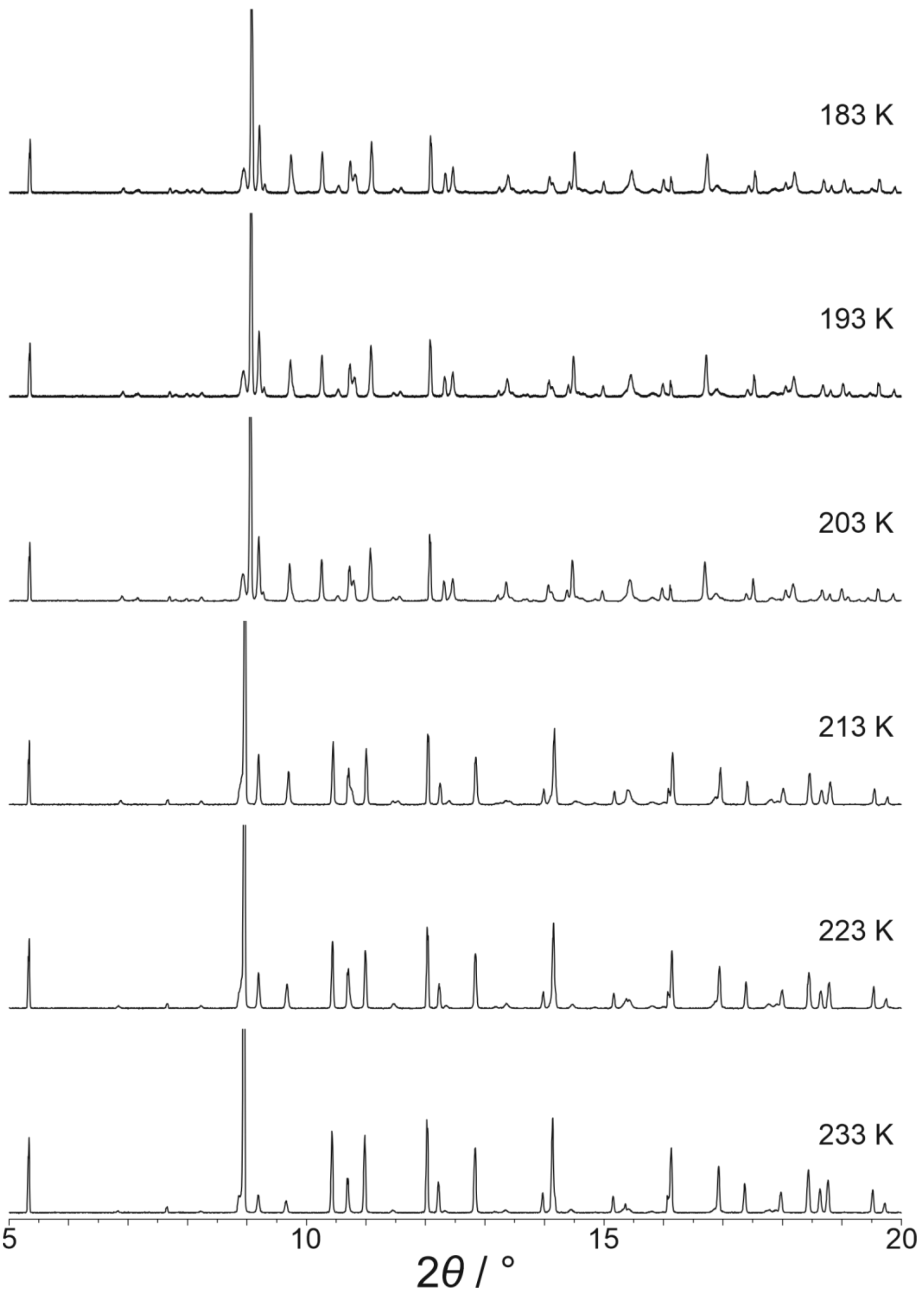

Figure S7: Powder XRD data recorded for 1-IA on cooling from 233 K to 183 K.

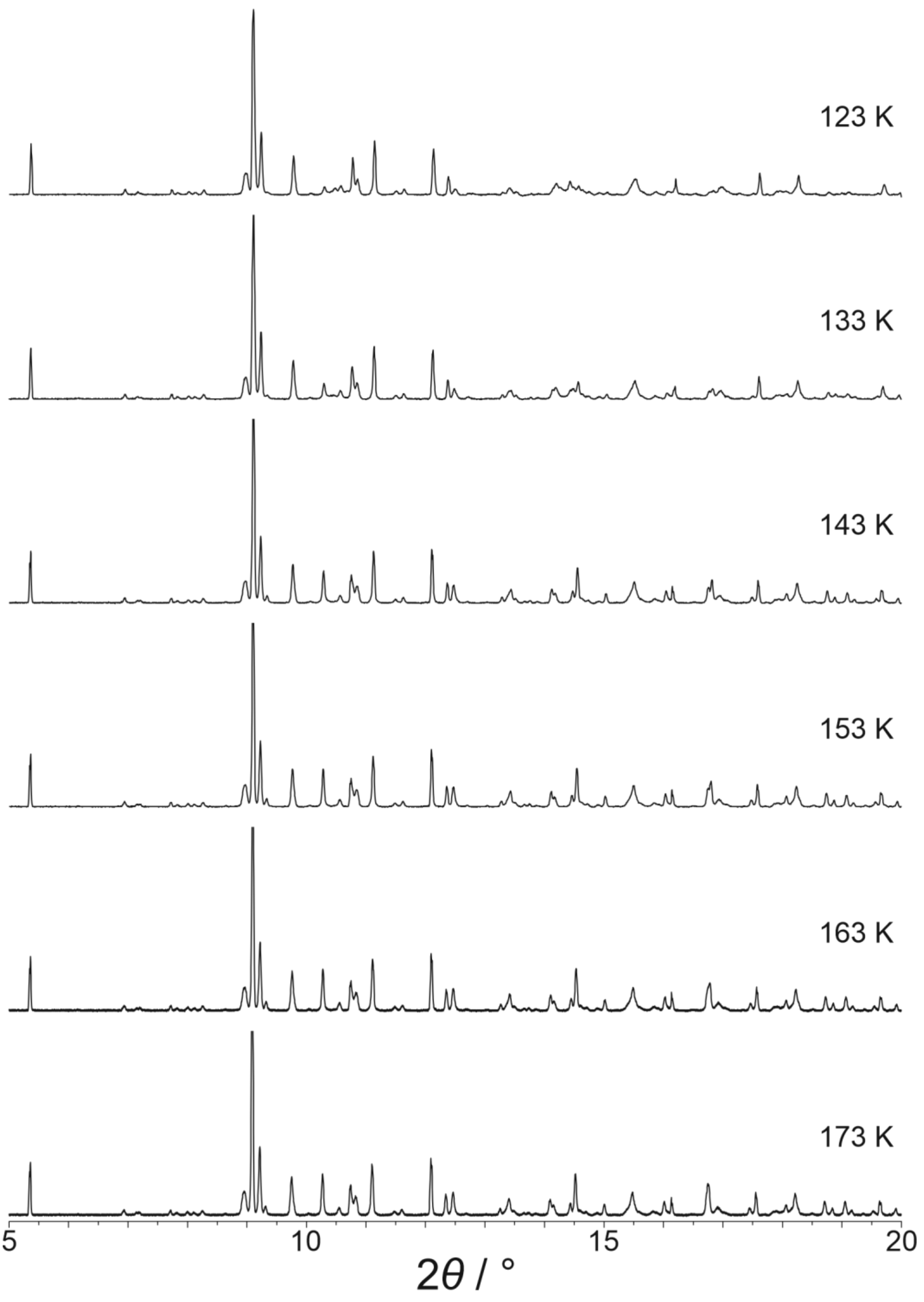

Figure S8: Powder XRD data recorded for 1-IA on cooling from 173 K to 123 K.

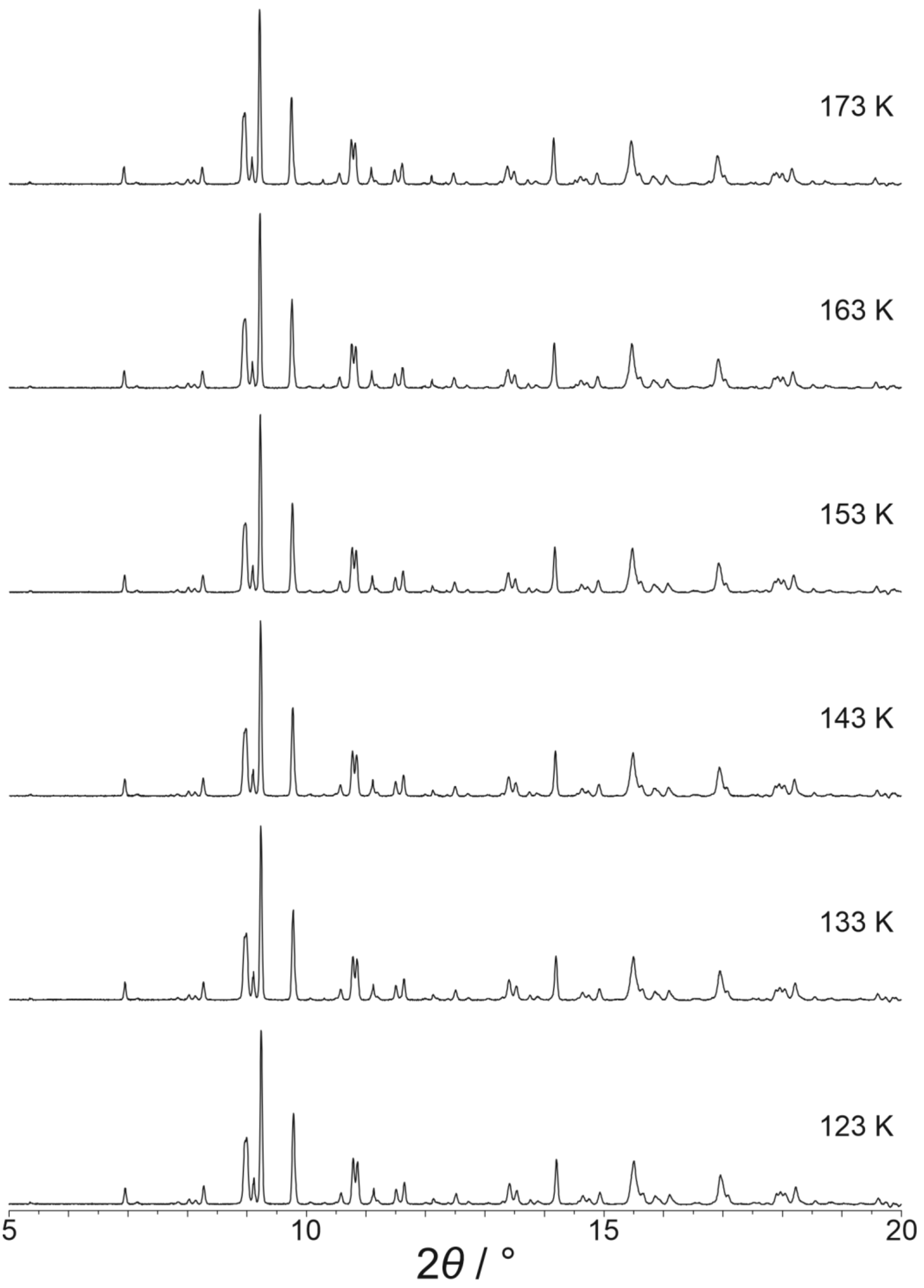

Figure S9: Powder XRD data recorded for 1-IA on heating from 123 K to 173 K.

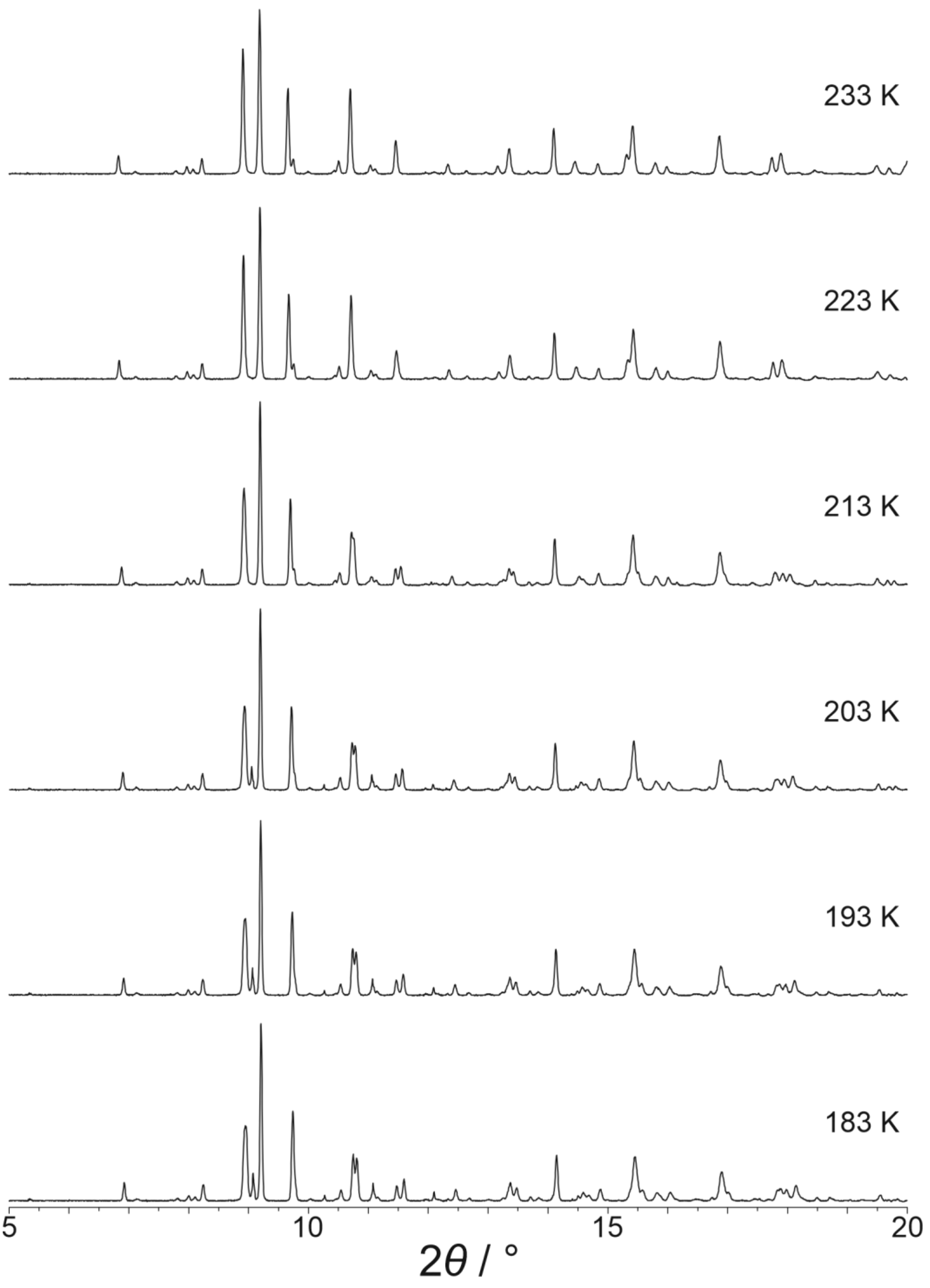

Figure S10: Powder XRD data recorded for 1-IA on heating from 183 K to 233 K.

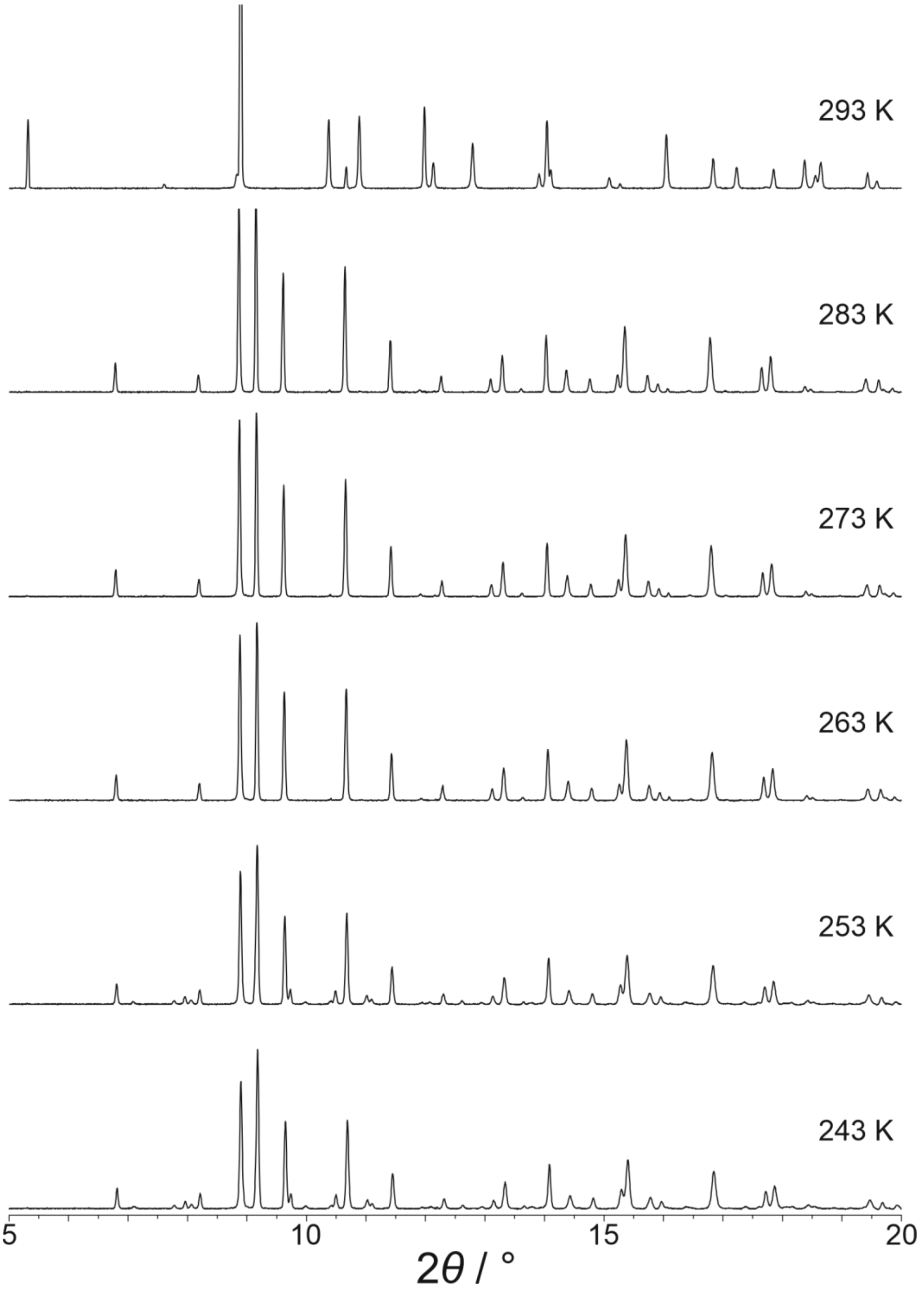

Figure S11: Powder XRD data recorded for 1-IA on heating from 243 K to 293 K.

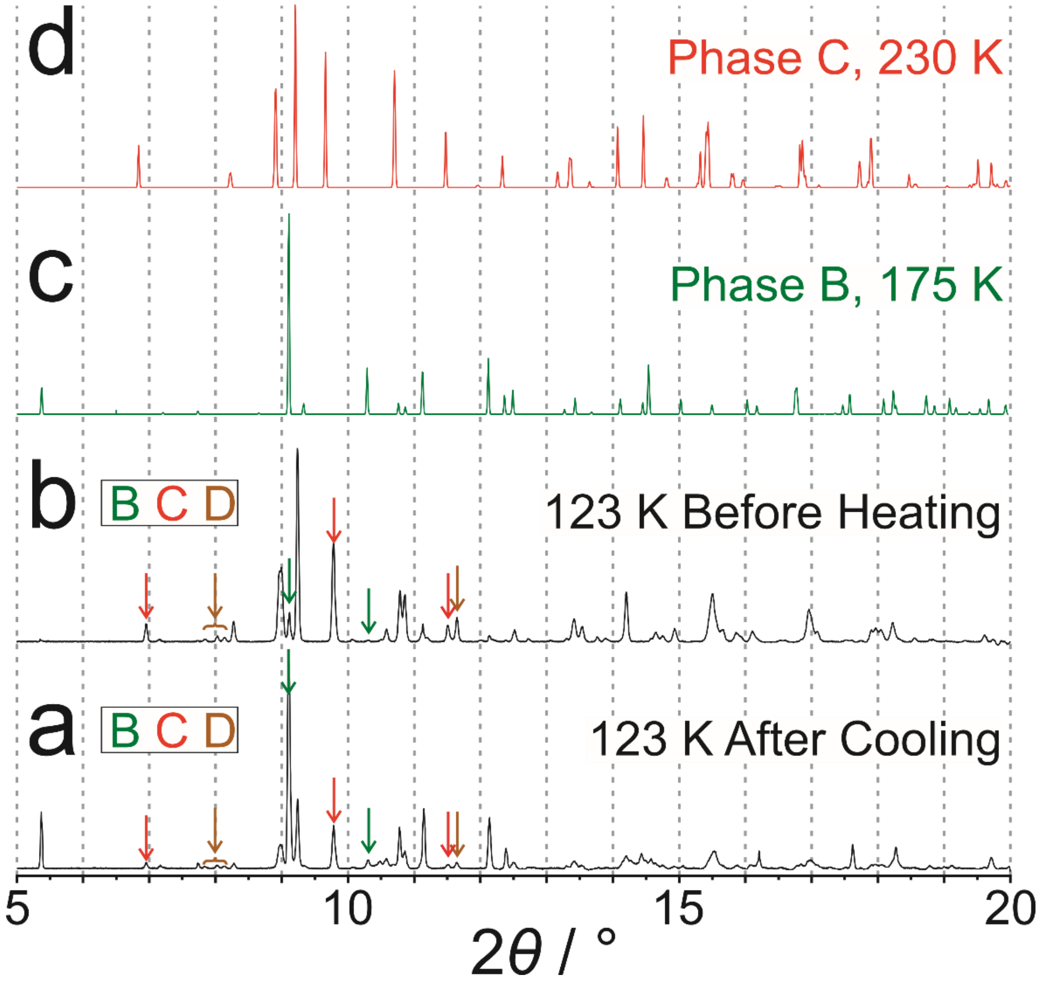

Figure S12. Powder XRD data recorded for 1-IA at 123 K (a) at the end of the cooling cycle and before moving the sample capillary, and (b) after moving the sample capillary and before commencing the heating cycle. Peaks indicated by arrows are diagnostic peaks for phase **B** (green arrows), phase **C** (red arrows) and phase **D** (brown arrows). In both (a) and (b), the powder XRD data indicate that the sample is a mixture of phases **B**, **C** and **D**, but with significantly different relative amounts of these phases, as discussed in the main paper. Powder XRD patterns simulated for the known crystal structures of (c) phase **B** (green) and (d) phase **C** (red) are also shown.

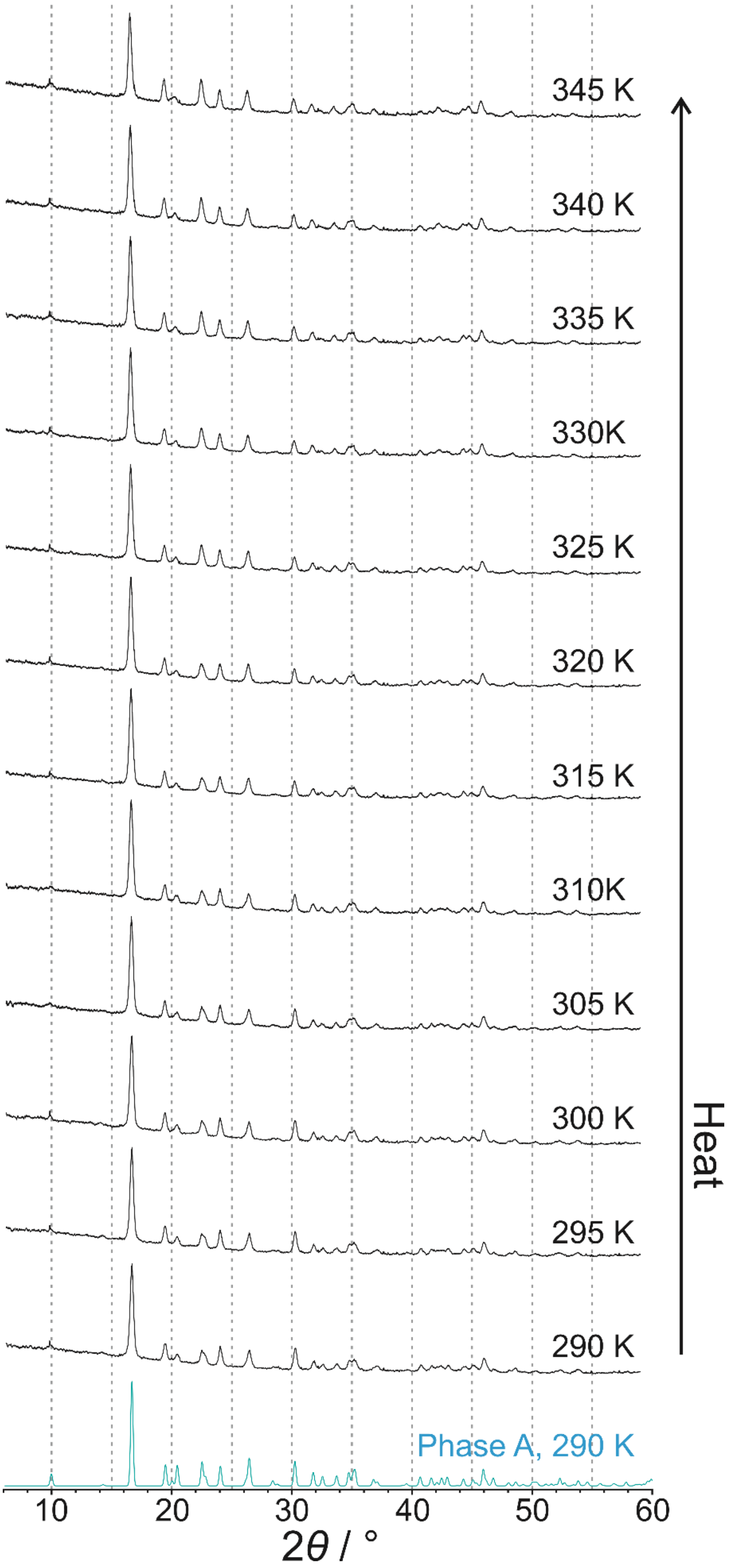

Figure S13. Powder XRD data recorded (using a laboratory powder XRD set-up) on heating a monophasic sample of phase **A** from ambient temperature (290 K) to a temperature (345 K) just below the melting temperature (348 K). The powder XRD pattern simulated for the known crystal structure of phase **A** (cyan) is also shown.

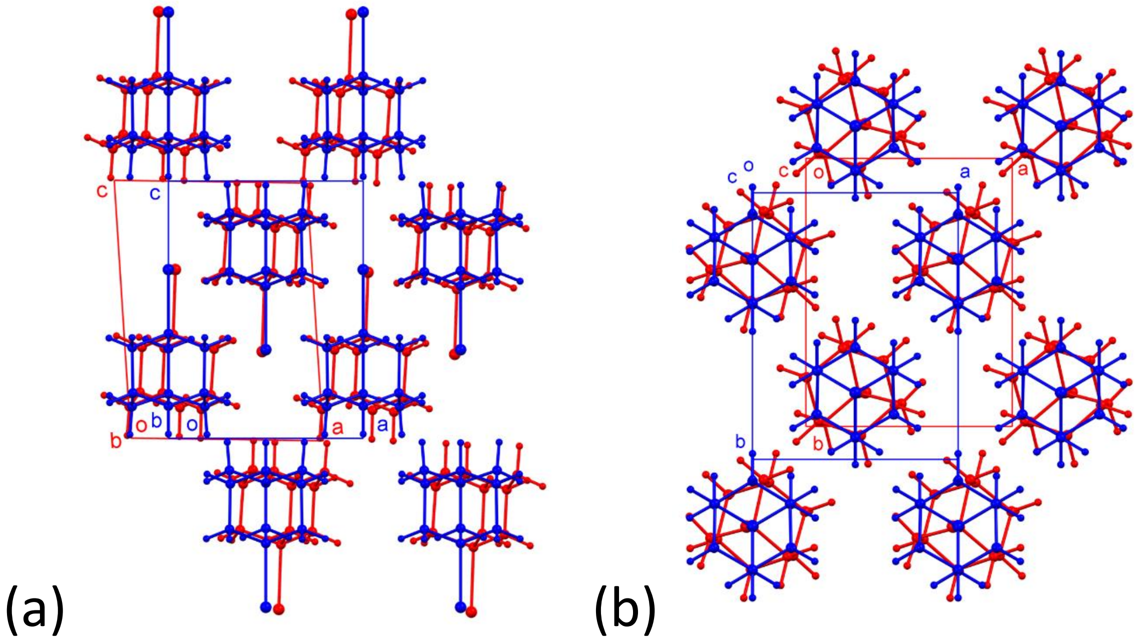

Figure S14. Overlay of the crystal structures of phase **A** (230 K; blue) and phase **B** (175 K; red) viewed along (a) the *b*-axis and (b) the *c*-axis, showing that there are only minor differences between these structures.

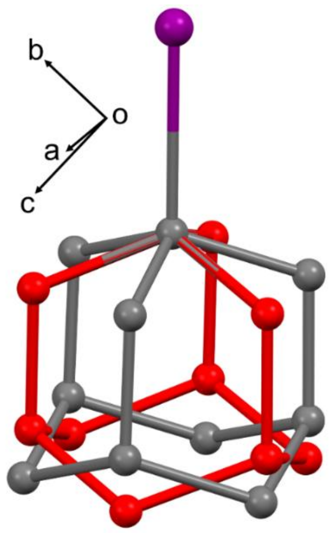

Figure S15. Orientational disorder of the 1-IA molecule in the crystal structure of phase **C** (determined for crystal **7** at 230 K; see Table S1). The disorder involves two orientations of the molecule related by 60° rotation about the C–I bond axis, with essentially equal occupancies of the two orientations. Hydrogen atoms are omitted for clarity.

Table S1: Crystallographic data from all eight single-crystal XRD studies to investigate changes in the structural properties of 1-IA as a function of temperature on cooling, starting from phase **A**. At temperatures marked with an asterisk, only unit cell determination was carried out. In all other cases, full structure determination was carried out. In the structure refinements for phase **C**, the refined occupancies of the two disorder components are: (i) 0.497(9)/0.503(9), (ii) 0.42(6)/0.58(2), (iii) 0.50(1)/0.50(1), (iv) 0.46(2)/0.54(2).

	T **/ K**	***a*** **/ Å**	***b*** **/ Å**	***c*** **/ Å**	***β*** **/ °**	***V*** **/ A³**	**Phase**	**R1**
**1**	290	6.7196(15)	8.6809(19)	8.8651(14)	90	517.12(18)	**A**	0.0542
	250	6.6940(13)	8.6190(16)	8.8506(11)	90	510.64(15)	**A**	0.0499
	240	6.6860(12)	8.6081(15)	8.8459(10)	90	509.11(14)	**A**	0.0494
	230	6.6786(12)	8.6080(12)	8.8296(10)	90	507.61(13)	**A**	0.0536
	220	6.6738(13)	8.5972(13)	8.8270(11)	90	506.46(14)	**A**	0.0497
	210	6.6117(16)	8.557(2)	8.8331(16)	94.01(2)	498.55(19)	**B**	0.0802
	175	6.5889(11)	8.5218(13)	8.8283(13)	94.438(14)	494.22(13)	**B**	0.0518
**2**	260	6.6949(14)	8.6470(11)	8.8462(9)	90	512.11(14)	**A**	0.0381
	230	6.6829(13)	8.6033(10)	8.8382(10)	90	508.15(13)	**A**	0.0350
	220	6.6813(12)	8.5970(10)	8.8392(10)	90	507.77(12)	**A**	0.0343
	215	6.6738(11)	8.5811(10)	8.8313(8)	90	505.76(11)	**A**	0.0356
	210	6.6150(12)	8.5439(11)	8.8503(11)	94.223(14)	498.84(13)	**B**	0.0477
**3**	280	6.6801(19)	8.6541(15)	8.8559(14)	90	511.96(19)	**A**	0.0379
	240	6.6614(18)	8.6062(14)	8.8427(13)	90	506.95(18)	**A**	0.0362
	175	6.5720(14)	8.5046(14)	8.8260(14)	94.538(17)	491.76(15)	**B**	0.0669
**4**	240	6.6817(12)	8.6063(12)	8.8245(7)	90	507.45(12)	**A**	0.1031
	220	6.6682(12)	8.5843(13)	8.8177(8)	90	504.74(13)	**A**	0.0832
	180	6.5857(11)	8.5219(10)	8.8294(8)	94.438(12)	494.04(11)	**B**	0.1420
	135	6.5550(11)	8.4861(13)	8.7991(13)	94.496(14)	487.96(13)	**B**	0.0948
**5**	290*	6.54(3)	8.59(4))	8.83(3)	90	496(4)	**A**	–
	175	6.5913(15)	8.5256(15)	8.8486(15)	94.65(2)	495.61(17)	**B**	0.0579
**6**	280*	6.713(4)	8.684(4)	8.831(11)	90	514.8(7)	**A**	–
	230	6.6773(16)	8.5893(16)	8.8381(16)	90	506.89(18)	**A**	0.0411
	200	6.6064(10)	8.5398(12)	8.8437(12)	94.365(14)	497.49(12)	**B**	0.1260
	120	6.5609(11)	8.4869(17)	8.8230(15)	94.676(17)	489.64(15)	**B**	0.0755
**7**	290*	6.676(15)	8.71(2)	8.851(15)	90	515(2)	**A**	–
	280	6.6994(11)	8.6631(12)	8.8546(7)	90	513.90(12)	**A**	0.0607
	230(i)	10.278(3)	6.9402(16)	13.826(3)	90.00(2)	986.2(4)	**C**	0.0904
	175(ii)	10.264(3)	6.9004(11)	13.619(3)	90.25(2)	964.5(3)	**C**	0.1522
**8**	280	6.7038(10)	8.6659(13)	8.8545(6)	90	514.40(11)	**A**	0.0433
	230	6.6808(16)	8.6173(17)	8.843(3)	90	509.1(2)	**A**	0.0591
	230(iii)	10.285(3)	6.9270(10)	13.8238(15)	90.00(2)	984.8(3)	**C**	0.0695
	175(iv)	10.2375(16)	6.8851(9)	13.594(2)	90.026(14)	958.2(2)	**C**	0.1294

Section S2: Difference Fourier Analysis of the Crystal Structure of Phase A

As noted in Section 2.5.1 of the main paper, the crystal structure of phase **A** reported previously by Foulon and Gors [Foulon, M.; Gors, C. *Acta Crystallogr. Sect. B* **1988**, *44*, 156-163] is described by space group Pmnn and exhibits disorder of each 1-IA molecule between two orientations differing by 60° rotation about the C–I bond axis. In contrast, analysis of single-crystal XRD data for phase **A** in the present work indicates that the correct space group is $Pm2_1n$, and the crystal structure does *not* exhibit disorder of the molecular orientation corresponding to 60° rotation about the C–I bond axis as reported by Foulon and Gors. Our conclusion that the crystal structure does not exhibit disorder of the molecular orientation corresponding to 60° rotation about the C–I bond axis is further supported by inspection of the difference Fourier electron density contour map (Figure S16) calculated at the plane $z = 0.644$, which cuts through atoms C2, C6 and C6'. The structural model used to calculate this difference Fourier map was based on the use of isotropic displacement parameters for C2 and C6/C6', recognizing that refinement of anisotropic displacement parameters for these atoms could potentially compensate for the presence of electron density in other molecular orientations arising from rotation around the C–I bond axis (which is perpendicular to the plane containing C2, C6 and C6'). The difference Fourier electron density contour map in Figure S16 does not show evidence for any significant residual electron density in the sites that would be occupied by C2, C6 and C6' in the molecular orientation corresponding to 60° rotation about the C–I bond axis. The highest peak (+0.9 e/$Å^3$) and lowest hole (–0.5 e/$Å^3$) in the vicinity of these atoms in the difference Fourier map are located *ca*. 0.5 Å from C2, C6 and C6', suggesting that they represent the effects of small-amplitude librational motion around the C–I bond axis rather than representing a significant population of the 1-IA molecule in the molecular orientation corresponding to 60° rotation about the C–I bond axis.

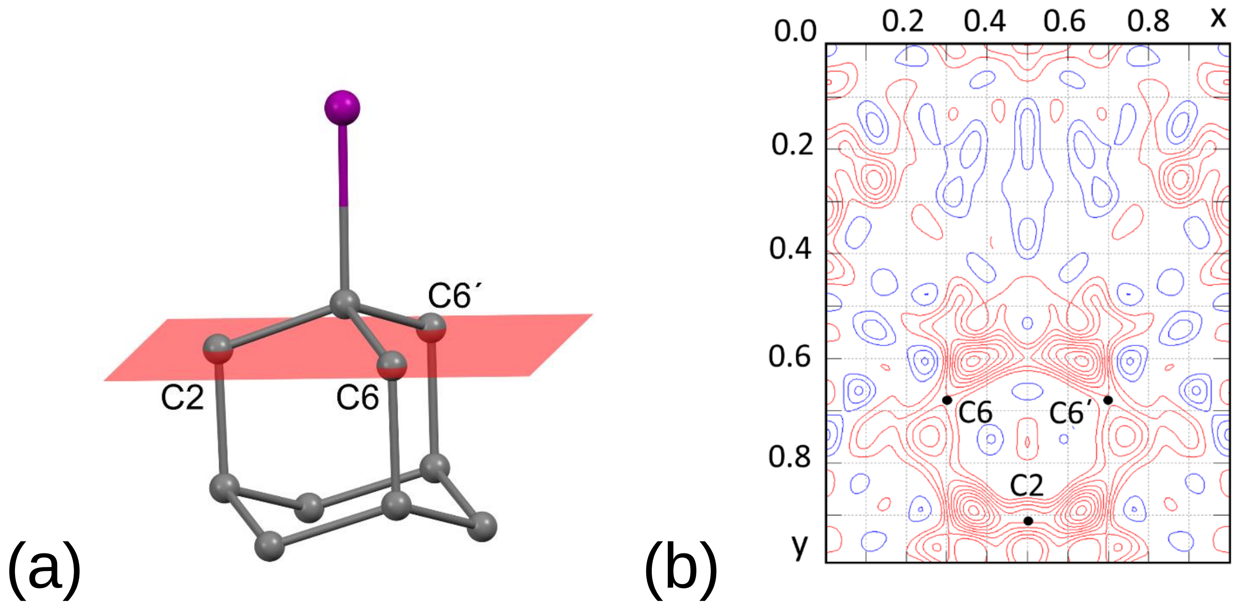

Figure S16. (a) Definition of the plane through atoms C2, C6 and C6' (at $z = 0.644$) in the crystal structure of phase **A** of 1-IA at 290 K. (b) Calculated difference Fourier electron density map at the plane through C2, C6 and C6' in the crystal structure (peaks are shown in red; holes are shown in blue). The magnitudes of the highest peak and lowest hole close to C2, C6 and C6' are +0.9 e/Å^3 and –0.5 e/Å^3, respectively.
